# Influenza-associated disease burden in mainland China: a systematic review and meta-analysis

**DOI:** 10.1038/s41598-021-82161-z

**Published:** 2021-02-03

**Authors:** Jing Li, Yinzi Chen, Xiling Wang, Hongjie Yu

**Affiliations:** 1 School of Public Health, Key Laboratory of Public Health Safety, Ministry of Education, Fudan University, Xuhui District, Shanghai, 200231 China; 2 Shanghai Key Laboratory of Meteorology and Health, Shanghai, China

**Keywords:** Influenza virus, Infectious diseases

## Abstract

Influenza causes substantial morbidity and mortality. Many original studies have been carried out to estimate disease burden of influenza in mainland China, while the full disease burden has not yet been systematically reviewed. We did a systematic review and meta-analysis to assess the burden of influenza-associated mortality, hospitalization, and outpatient visit in mainland China. We searched 3 English and 4 Chinese databases with studies published from 2005 to 2019. Studies reporting population-based rates of mortality, hospitalization, or outpatient visit attributed to seasonal influenza were included in the analysis. Fixed-effects or random-effects model was used to calculate pooled estimates of influenza-associated mortality depending on the degree of heterogeneity. Meta-regression was applied to explore the sources of heterogeneity. Publication bias was assessed by funnel plots and Egger’s test. We identified 30 studies eligible for inclusion with 17, 8, 5 studies reporting mortality, hospitalization, and outpatient visit associated with influenza, respectively. The pooled influenza-associated all-cause mortality rates were 14.33 and 122.79 per 100,000 persons for all ages and ≥ 65 years age groups, respectively. Studies were highly heterogeneous in aspects of age group, cause of death, statistical model, geographic location, and study period, and these factors could explain 60.14% of the heterogeneity in influenza-associated mortality. No significant publication bias existed in estimates of influenza-associated all-cause mortality. Children aged < 5 years were observed with the highest rates of influenza-associated hospitalizations and ILI outpatient visits. People aged ≥ 65 years and < 5 years contribute mostly to mortality and morbidity burden due to influenza, which calls for targeted vaccination policy for older adults and younger children in mainland China.

## Introduction

Seasonal influenza circulates annually and causes substantial morbidity and mortality, with the highest burden among adults aged ≥ 65 years and children aged < 5 years. Every year it causes an estimated 3 to 5 million cases of severe illness and 290,000–650,000 respiratory deaths throughout the world^1^. The majority of influenza-related deaths occur in adults aged ≥ 65 years, accounting for over 85% of deaths^2–6^. Influenza is associated with 610,000–1,237,000 respiratory hospitalizations in children aged < 5 years worldwide annually^7^.

A direct way to measure disease burden of influenza is usually based on syndromic surveillance for influenza. A 2018 systematic review and meta-analysis by Shang et al.^8^ estimated the laboratory-confirmed respiratory hospitalizations attributed to influenza among children aged < 18 years in China. Considering under-ascertainment and under-reporting of influenza, indirect statistical modeling method provides more accurate estimates of influenza burden than those solely relying on laboratory-confirmed influenza case counts. Three nationally representative studies have estimated the influenza-associated mortality rates and ILI outpatient visit rates in mainland China by modeling techniques^9–11^. A 2019 systematic review by Li et al.^12^ described the influenza-associated mortality rates in mainland China by age group, cause of death, geographic location, and influenza virus type/subtype, but it did not produce synthesized estimates.

Although many studies have been conducted in China to estimate disease burden of seasonal influenza, the findings vary across different study periods, regions, populations, circulating virus strains, and methodologies^10,13,14^. There is no quantitatively synthesized estimate available so far to guide national influenza prevention strategies and healthcare resource allocation. Our study aims to systematically review the Chinese and English literature of influenza-associated mortality, hospitalization, and outpatient burden in mainland China.

## Methods

### Search strategy

We followed the Preferred Reporting Items for Systematic reviews and Meta-Analyses (PRISMA) guidelines^15^ to conduct this review. Firstly, we searched 7 electronic databases (PubMed, EMBASE, Web of Science, CNKI [China National Knowledge Infrastructure], Wan-Fang Data, Chong-Qing VIP, and CBM [China Biology Medicine disc]) to identify relevant studies published from 1 January 2005 to 31 December 2019. A search strategy was developed and adapted for each database with a combination of search terms in “all fields” and “title/abstract”. Keywords included: “influenza”, “burden”, “mortality”, “hospitalization”, “outpatient”, “China”, and other designators to indicate the contribution of influenza, such as “excess”, “associated”, “related”, “attributed”, “attributable”, “confirmed”. A full list of search terms and results was provided in Table S1. Secondly, reference lists of eligible studies, as well as the reference lists of reviews, were manually checked for additional studies. Thirdly, we searched additional electronic sources including Google Scholar (https://scholar.google.com/) and Baidu Scholar (http://xueshu.baidu.com/). Titles and abstracts of the unique articles were screened by two reviewers (J.L. and Y.C.) independently. Full texts of all potentially eligible articles were retrieved and screened for final inclusion. Discrepancies in the study selection between the two reviewers were resolved by consensus or the involvement of a third reviewer (X.W.).

### Inclusion criteria

We included published peer-reviewed articles in English and Chinese language. We constrained the starting year from 2005 because the influenza surveillance network became much more robust in China which was launched in 2000. Eligible articles were those reporting population-based rates of mortality, hospitalization, and outpatient visit attributed to seasonal influenza, using statistical modeling or laboratory-confirmed methods in mainland China. We included 6 most commonly used causes of death, coded according to the International Classification of Diseases 10th Revision (ICD-10), which could be attributed to influenza infection: (1) all-cause (ICD-10: A00-A99); (2) respiratory and circulatory disease (ICD-10: J00-J99, I00-I99); (3) respiratory disease (ICD-10: J00-J99); (4) pneumonia and influenza (ICD-10: J09-J18); (5) chronic obstructive pulmonary disease (ICD-10: J40-J47); (6) ischemic heart disease (ICD-10: I20-I25). Considering that studies of influenza morbidity burden were few in China, we extracted rates as reported in the studies.

### Exclusion criteria

Studies were excluded if: (1) they were prediction studies; (2) only reporting estimates for a specific risk population, such as pregnant women or individuals with chronic medical conditions; (3) only reporting estimates for China’s special administrative regions (SARs); (4) the study period was less than a year or an influenza season; (5) population-based estimates of influenza burden could not be derived; (6) duplicate estimates, such as reporting estimates on a subset of published data or previous estimates that have been updated using the latest data; (7) they were reviews, conference proceedings, commentaries, editorials, and letters. Studies with a time frame combining both seasonal and pandemic influenza period were included, but we excluded the estimates for the 2009 H1N1 pandemic.

### Data extraction

Data were extracted into a predefined form by two reviewers independently and in duplicate (J.L. and Y.C.). For each included study, the following data were extracted: general characteristics of the studies, methodological characteristics, and primary outcome measures. The primary outcomes included: annual average influenza-associated mortality rates by age group (all ages, < 65 years, and ≥ 65 years) and cause of death; annual average influenza-associated hospitalization rates and outpatient visit rates by age group and case definition/discharge diagnosis code. If the burden estimates were reported by year, we calculated the average estimates directly. For studies only reporting the number of excess deaths, we calculated the excess rates by dividing excess numbers to the population sizes. Statistical models used to estimate influenza burden were classified as multiplier method, Serfling model, rate difference model, and regression model with an influenza activity proxy (including Poisson model, negative binomial model, and linear model). Geographic locations were defined as northern or southern China (demarcated by the Qinling Mountains-Huaihe River line^8^).

### Quality assessment

As there was no existing appraisal tool for modeling studies, we modified Bhuia et al.’s developed checklist which had been used to assess models for estimating disease burden of asthma^16^. Laboratory-confirmed studies were assessed using the JBI Critical Appraisal Checklist for Case Series^17^. Both checklists were consisted of 10 items with a maximum score of 10 (Tables S2, S3). Studies with scores of 8 or greater were considered as high-quality studies. We evaluated the quality of the evidence for mortality outcome using the Grading of Recommendations Assessment, Development and Evaluation (GRADE) tool^18^. The GRADE of observational studies is rated as low certainty of evidence. This rating can be downgraded by five domains (risk of bias, inconsistency, indirectness, imprecision, and publication bias) and can be upgraded by three domains (large magnitude of effect, dose response, and attenuation by plausible confounding).

### Statistical analysis

Meta-analyses were conducted to pool the influenza-associated mortality rates and 95% confidence intervals (CIs) when 5 or more estimates were reported. To examine the age-specific estimates of influenza-associated mortality rates across different causes of death, we stratified the meta-analyses by cause of death and age group. Statistical heterogeneity of the estimates was assessed using Cochran’s *Q* test and ${I}^{2}$ statistic^19^. If ${I}^{2}$ statistic was above 50% or *P* value of *Q* statistic was lower than 0.10, the estimates were pooled by random-effects model; otherwise, fixed-effects model was used. Standard errors (SEs) were used in the calculation of the precision of the pooled mortality estimates. If SEs cannot be directly derived from the 95% CIs, they were imputed by linear regression model using excess mortality rate, age group, and cause of death as predictors with a log-linked function^20^. The pooled estimates were standardized using the age structure of the 2010 population census of China^21^ as the reference.

To explore the sources of heterogeneity in estimates of influenza-associated mortality rates, subgroup analyses and meta-regression analysis were performed by the following variables: age group, cause of death, statistical model, geographic location, and study period. The ${R}^{2}$ statistic was used to indicate the proportion of total variance explained by the covariates examined. Sensitivity analysis was conducted by excluding studies with quality scores below 8 in the meta-analyses. Publication bias was assessed by funnel plots and Egger’s test^22^.

Meta-analyses on influenza-associated hospitalization and outpatient burden were not performed due to the paucity of studies. As the burden estimates may differ in urban and rural areas, Pearson correlation analysis was applied to explore the relationship between urbanization rate (urban population as a percentage of the total population) and influenza-associated mortality or morbidity.

This study was registered with PROSPERO, number CRD42019133748. The flow diagram was drawn using Adobe Illustrator software, version 22.0.0 (https://www.adobe.com/products/illustrator.html). All analyses and other figures were performed using R software, version 3.5.2 (https://www.r-project.org/).

## Results

### Study selection

**Figure 1:**
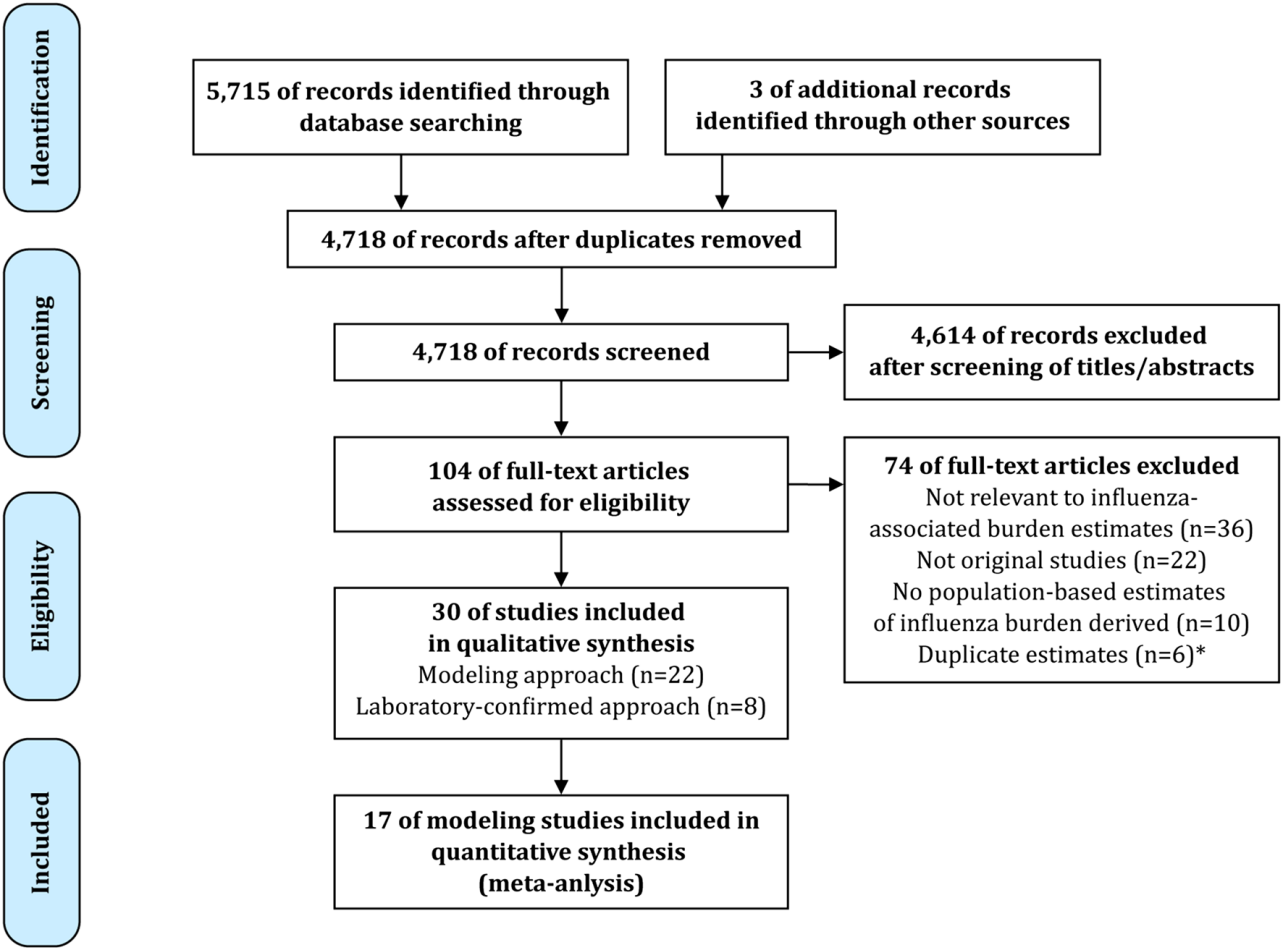
Flow diagram for systematic review process. *If several publications reported on the same study, we only included the publication that provided the most data. The flow diagram was drawn using Adobe Illustrator software, version 22.0.0 (https://www.adobe.com/products/illustrator.html).

We identified 5,715 records through database searching and added 3 additional records through review of reference lists, among which 4,718 records were unique after removal of duplicates (Fig. 1). 4,614 records were excluded after screening the titles and abstracts. 104 full-text articles were retrieved for further assessment. Of these, 74 articles were excluded due to the following reasons: not relevant to influenza-associated burden estimates (n = 36), not original studies (n = 22), no population-based estimates of influenza burden derived (n = 10), duplicate estimates (n = 6). Finally, 30 articles were eligible for the systematic review including 22 reporting modeling estimated influenza burden and 8 reporting laboratory-confirmed influenza burden. 17 studies reporting influenza-associated mortality using modeling approach were included in the meta-analysis.

### Study characteristics

**Figure 2:**
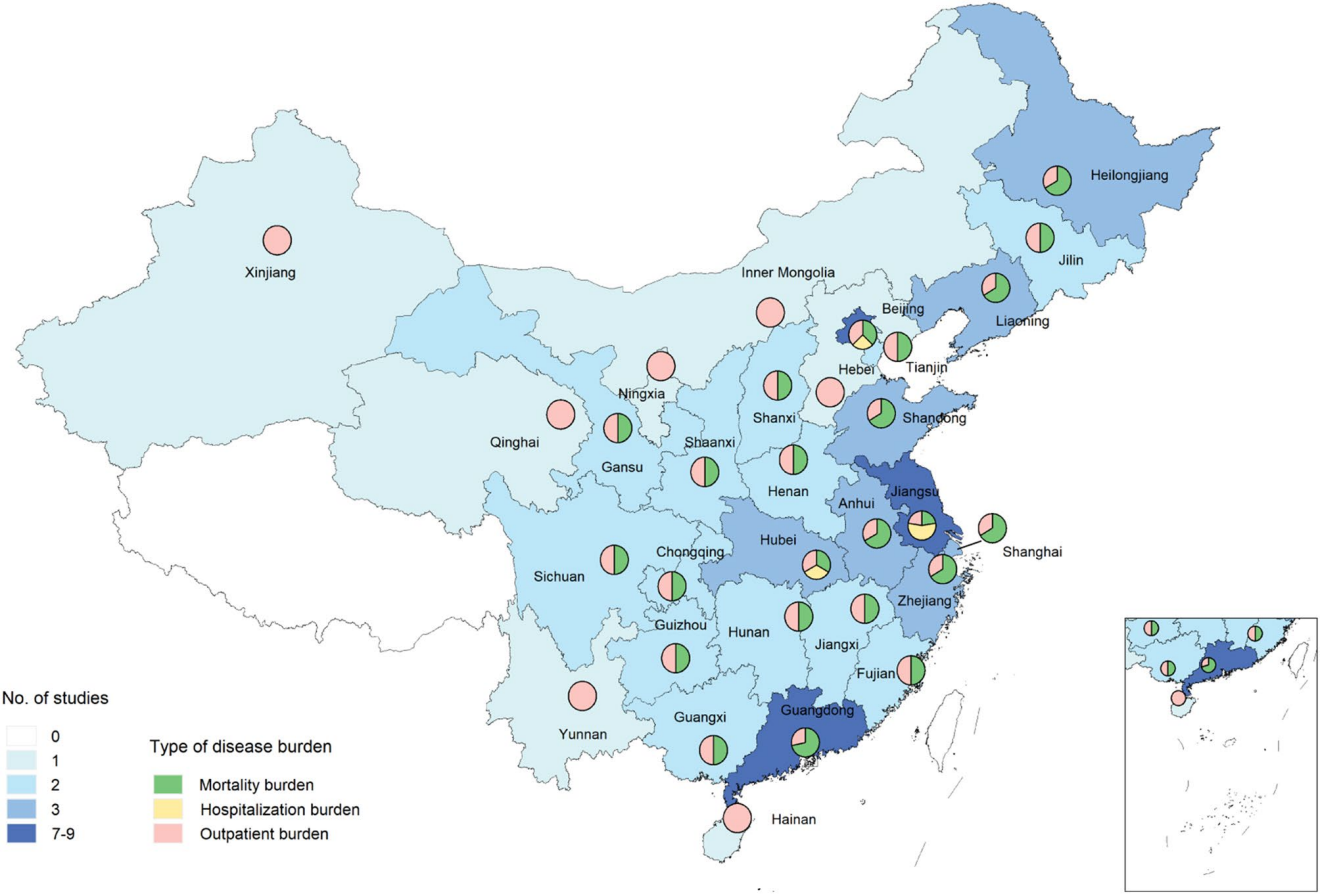
The distribution of published studies about influenza-associated disease burden. Two studies of mortality burden were not shown in the figure because they reported combined estimates of multiple cities^5^ or Disease Surveillance Points (DSP) sites^9^. One study could have estimated influenza disease burden for multiple provinces or cities. The map was drawn using R software, version 3.5.2 (https://www.r-project.org/).

Among the 30 included studies, 17 assessed influenza-associated mortality burden (all from modeling studies^5,9,10,13,14,24–34^), while 8 assessed influenza-associated hospitalization burden (2 from modeling studies^35,36^ and 6 from laboratory-confirmed studies^37–42^) and 5 assessed influenza-associated ILI outpatient burden (3 from modeling studies^11,43,44^ and 2 from laboratory-confirmed studies^45,46^) (Table S4). The study periods included pre-pandemic (n = 9), post-pandemic (n = 17), and both periods (n = 4). Lists of all included articles were provided in Tables S5 and S6. The 30 articles reported 84 regional estimates, mostly in the more developed eastern provinces, such as Jiangsu, Beijing, Guangdong, etc., consisting of 37 (44%) for northern China and 47 (56%) for southern China (Fig. 2).

The quality scores of included studies ranged from 7 to 10 points (Tables S7, S8). Most studies (25/30) were of high quality. Lack of model validation and no description of missing data were commonly seen in modeling studies, while no reporting of follow-up health outcomes was often observed in laboratory-confirmed studies.

Eighteen studies used regression models with an influenza activity proxy^5,9,10,11,13,14,24–29,31,33–36^; 5 studies applied Serfling models^5,23,27,32,34^; 2 studies used rate difference models^25,30^; and 2 applied multiplier methods^43,44^ (Table S4). Four different influenza activity proxies were used in modeling studies: positive number of laboratory-confirmed influenza (LAB number), positive proportion of laboratory-confirmed influenza (LAB%), influenza-like illness consultation rate (ILI%), and product of positive proportion of laboratory-confirmed influenza and influenza-like illness consultation rate (LAB% $\times$ ILI%). The most commonly used influenza activity proxy (14/19) was LAB%. Smoothing function of time^10,11,14,24,28,31,33^ or the combination of linear, quadratic, cubic, and trigonometric functions of time^5,9,13,23,26,27,29,32,34–36^ was commonly used to account for time trends in the regression models. Some studies also included meteorological factors, such as absolute humidity only^14,31^ or the combination of temperature and absolute/relative humidity^10,11,24,28,33^ as confounders. Six of the 8 laboratory-confirmed studies used reverse transcription-polymerase chain reaction (RT-PCR) testing methods to confirm influenza from patient samples^38–41,45,47^, and the remaining used immunofluorescence assay (IFA) (n = 1)^42^ and hemagglutination inhibition assay (HI) (n = 1)^46^ (Table S4). Three case definitions were used to identify patients with severe acute respiratory infection (SARI) (n = 5)^38–41,47^, pneumonia (n = 1)^42^ or influenza-like illness (ILI) (n = 2)^45,46^. Details of the case definitions used in the laboratory-confirmed studies were shown in Table S6.

### Influenza-associated mortality burden

#### Pooled estimates by cause of death and age group

Pooled influenza-associated all-age mortality rates per 100,000 persons were as follows: all-cause, 14.33 (95% CI 11.56, 17.10); respiratory and circulatory disease, 10.89 (8.71, 13.07); respiratory disease, 5.84 (4.66, 7.03); pneumonia and influenza, 0.67 (0.27, 1.07); chronic obstructive pulmonary disease, 2.66 (1.50, 3.83); ischemic heart disease, 2.57 (0.97, 4.18) (Table 1). The pooled rates of influenza-associated all-cause and cause-specific deaths for adults aged ≥ 65 years were significantly higher than that for those aged < 65 years. Heterogeneity increased with age in the pooled influenza-associated mortality rates, from 0.0 to 53.6% for < 65 years to 97.5–99.7% for ≥ 65 years (Figs. S1–S6).

**Table 1:** Pooled influenza-associated mortality rates per 100,000 persons by cause of death and age group.

Outcome	No. of estimates	Influenza-associated mortality rates (95% CI)
**All-cause**		
All ages	23	14.33 (11.56, 17.10)
Age-standardized	12	14.76 (11.54, 17.98)
< 65 years	12	2.67 (2.27, 3.07)
≥ 65 years	17	122.79 (92.23, 153.34)
**Respiratory and circulatory disease**		
All ages	20	10.89 (8.71, 13.07)
Age-standardized	12	11.01 (8.77, 13.26)
< 65 years	12	1.55 (1.30, 1.80)
≥ 65 years	16	95.29 (71.67, 118.92)
**Respiratory disease**		
All ages	32	5.84 (4.66, 7.03)
Age-standardized	27	5.03 (3.91, 6.16)
< 65 years	27	1.23 (0.64, 1.82)
≥ 65 years	28	43.71 (34.33, 53.09)
Pneumonia and influenza*		
All ages	12	0.67 (0.27, 1.07)
≥ 65 years	10	8.73 (1.10, 16.35)
Chronic obstructive pulmonary disease*		
All ages	8	2.66 (1.50, 3.83)
≥ 65 years	5	20.67 (5.08, 36.26)
Ischemic heart disease*		
All ages	7	2.57 (0.97, 4.18)
≥ 65 years	5	19.15 (6.97, 31.33)

*Due to the small numbers (< 5) of the reported estimates for age-standardized and < 65 years, the meta-analyses were not performed.

#### Sources of heterogeneity

The subgroup analyses found that influenza-associated mortality rates were different with statistical significance by age group and cause of death, while statistically insignificant by statistical model, geographic location, and study period (Table S9). When these variables were entered into the meta-regression, it yielded a significant model (*F* $=$ 26.40, *P* $<$ 0.001), that explained 60.14% of the heterogeneity in the estimates. The variation was significantly associated with age group, cause of death, and statistical model (Table 2). Estimates of influenza-associated mortality rates were higher for all ages and ≥ 65 years than for < 65 years and were higher with broader cause-of-death groups (Table 2, Fig. S7). Studies using rate difference models were generally associated with higher estimates than other models. The estimates were higher from southern China than from northern China and were higher in the pre-pandemic period than in the post-pandemic period, but the differences did not reach statistical significance (Table 2, Fig. S8). No strong correlation was observed between urbanization rates and influenza-associated mortality rates (Fig. S9).

**Table 2:** Results of a meta-regression analysis conducted to identify factors that influence estimates of influenza-associated mortality.

Variable	Influenza-associated mortality rates per 100,000 persons (95% CI)	*P* value
**Age group**		
All ages	5.93 (− 2.41, 14.28)	0.163
< 65 years	Reference	
≥ 65 years	58.95 (50.15, 67.74)	< 0.001
**Cause of death**		
All-cause	43.74 (30.93, 56.54)	< 0.001
Respiratory and circulatory disease	34.53 (21.56, 47.49)	< 0.001
Respiratory disease	17.38 (5.62, 29.14)	0.004
Pneumonia and influenza	Reference	
Chronic obstructive pulmonary disease	6.01 (− 10.54, 22.55)	0.475
Ischemic heart disease	6.01 (− 10.45, 22.48)	0.473
**Statistical model**		
Serfling model	Reference	
Rate difference model	20.25 (3.53, 36.98)	0.018
Regression model with an influenza activity proxy	10.14 (− 0.56, 20.85)	0.063
**Geographic location**		
Northern China	Reference	
Southern China	0.51 (− 6.70, 7.72)	0.889
**Study period**		
Pre-pandemic	4.42 (− 3.54, 12.38)	0.275
Post-pandemic	Reference	
Both	0.13 (− 22.60, − 22.87)	0.991

#### Sensitivity analyses, publication bias, and GRADE

Our results were robust as the sensitivity analysis showed that the pooled estimates were similar to the main analysis (Table S10). No significant publication bias was found in most cause- and age-specific influenza-associated mortality estimates (Fig. S10), except for respiratory disease (all ages: *P* $=$ 0.024, age-standardized: *P* $=$ 0.001, and < 65 years: *P* $=$ 0.042) and pneumonia and influenza (≥ 65 years: *P* $=$ 0.005). The upgrade domains were not applicable to time series modeling studies. Beginning with a low-certainty rating, the certainty of evidence was downgraded by one level for most cause- and age-specific influenza-associated mortality rates, owing to inconsistency (${I}^{2}>$ 50%, *P* $<$ 0.001).

### Influenza-associated hospitalization burden

Studies reporting influenza-associated hospitalization burden were conducted in Jiangsu (n = 5), Beijing (n = 2), and Jingzhou (n = 1). Two studies used statistical modeling methods to estimate influenza-associated P&I hospitalization rates among children aged < 5 years (384 per 100,000 children)^35^ and < 15 years (112 per 100,000 children)^36^ in Jiangsu. Five of the 6 laboratory-confirmed hospitalization studies used SARI definition to screen inpatients^37–41,47^, while the remaining study used pneumonia case definition^42^. The influenza-associated SARI hospitalization rate in children aged < 5 years ranged from 442 in Beijing to 715 in Suzhou per 100,000 persons^38–41,47^, except that Jingzhou reported 2185 per 100,000 persons because of a lower fever threshold in case definition^37^ (Table S11).

### Influenza-associated outpatient burden

A national study provided estimates of influenza-associated ILI outpatient visit rates from 2006 to 2015 across 30 provinces, ranging from 0.1 in Ningxia to 7.4 in Shanghai per 1000 persons^11^. The highest influenza-associated outpatient visit rate was found in the age group < 15 years. We identified a significant positive correlation between urbanization rates and influenza-associated ILI outpatient visit rates across these provinces (r $=$ 0.85, *P* $<$ 0.001) (Fig. S9). Two studies using multiplier methods were conducted in Beijing and reported rates of influenza-associated ILI outpatient visits for the 2015–2016 season (22 per 1,000 persons) and 2017–2018 season (69 per 1000 persons), with the highest rates observed in children aged < 5 years (154 and 330 per 1000 persons respectively)^43,44^. For the remaining 2 laboratory-confirmed studies, influenza-associated ILI outpatient visit rates among children < 5 years ranged from 34 per 1000 persons in Zhuhai to 64 per 1000 persons in Suzhou^45,46^.

## Discussion

Our study systematically reviewed influenza-associated disease burden studies in mainland China, including estimates both from modeling approach and laboratory-confirmed approach. Mortality burden of influenza was assessed not only for respiratory deaths but also for circulatory deaths, which provided better understanding of the overall impact of influenza.

The subgroup analyses indicated that the estimates of influenza-associated mortality were significant only in age group and cause of death. However, when controlling for other variables in the meta-regression, statistical model also became significant. In our meta-analysis, influenza-associated mortality rates by cause of death and age group showed high levels of heterogeneity, which might be due to the variation in statistical models. Robust estimates indicated by sensitivity analysis and no publication biases demonstrate the stronger certainty of evidence than original observational studies included in our review. Our study also benefits from the strengths of original studies including data from well-established surveillance systems and generally high-quality studies.

In line with previous studies^6,48–50^, influenza-associated mortality rate was found the highest among adults aged ≥ 65 years. Our pooled influenza-associated respiratory mortality rate (5.84 per 100,000 persons) fell within the estimate for the Western Pacific region reported in a global study (3.6–7.5 per 100,000 persons)^2^. Our pooled estimates were also comparable with the corresponding estimates published for other countries or regions; e.g. our pooled excess all-cause mortality rate (14.33 per 100,000 persons) was similar to those reported in Europe (13.30 per 100,000 persons)^48^ and Hong Kong SAR (11.70 per 100,000 persons)^6^, but slightly higher than that reported in the USA (6.96 per 100,000 persons)^49^ and South Korea (5.97 per 100,000 persons)^50^. Our influenza-associated P&I (384 per 100,000 persons) or SARI (442–715 per 100,000 persons) hospitalization rate among children aged < 5 years was much higher than those reported for Singapore (excess P&I hospitalization rate of 186.8 per 100,000 persons)^51^, Portugal (excess P&I hospitalization rate of 42.6 per 100,000 persons)^52^, Chile (excess SARI hospitalization rate of 71.5 per 100, 000 persons)^53^. Children aged < 5 years had the highest excess SARI hospitalization rates in China, contrasting with higher rates among adults aged ≥ 65 years in above countries. However, it is difficult to make like-for-like comparisons between the countries’ estimates due to different healthcare seeking behavior or access to healthcare services. Lower mortality or morbidity rates in these countries could be partially explained by their higher vaccination coverage.

Influenza vaccination can reduce the risk of illness caused by influenza virus infection by about 40–60% among the general population^54^, while the vaccination coverage in the Chinese population was only approximately 2%^55^. The highest influenza disease burden found among people aged ≥ 65 years and < 5 years in China highlights the priority to strengthen vaccination strategy in these two age groups. Unlike many high-income countries, influenza vaccination is not included in the National Immunization Program in China. Only a few developed cities such as Beijing have provided free influenza vaccination for older adults using local revenue and have achieved a remarkable increase in local vaccine uptake^56^. In addition, a recent cost-effectiveness study suggests that government fully funded immunization against influenza among older adults proves to be cost-effective for influenza control and prevention in China^57^. Our findings collectively with this evidence inform immunization recommendations in China.

### Methodological issues

In our study, estimates of influenza-associated mortality rates from the Serfling models were lower than those from other models, which contradicts the conclusion of the previous review by Li, et al. that Serfling models were associated with higher estimates^20^. Serfling models would inherently overestimate the influenza burden because this approach attributes all excess deaths to influenza^58^. However, more conservative estimates were produced from Serfling models included in our study, primarily because only a small set of weeks were classified as epidemic weeks in calculating the influenza burden in the Serfling analysis after adding the restriction of “for 2 or more consecutive weeks”. The performance of different influenza activity proxies may differ in estimating the burden of influenza. Although LAB% was the most commonly used influenza activity proxy, ILI $\times$ LAB may be more closely related to influenza incidence^14^. The choices of which meteorological factors to include in the models are still controversial. Several studies have demonstrated the role of temperature, relative humidity, or absolute humidity on influenza virus survival and transmission^59,60^. Of note, collinearity might be considered when using the combination of temperature and absolute humidity because absolute humidity can be calculated based on temperature and relative humidity. RSV and other pathogen activity were not considered in all the regression models because there is currently no national surveillance for the co-circulating respiratory viruses in China, which may have confounded the results.

### Limitations

Our study had several limitations. Firstly, high levels of heterogeneity existed across influenza-associated mortality studies. Although we applied random-effect model to account for the heterogeneity, the pooled estimates from meta-analysis should be interpreted with caution as it might not represent the national burden. Secondly, we did not assess the impact of predominant virus types/subtypes and high-risk populations because studies were too few after stratification, which could also be sources of mortality variation. Thirdly, we used a regression model to estimate the missing SEs; thus, the difference between estimated and true SEs may have influenced the estimates of pooled influenza-associated mortality.

### Future directions

Our review found that influenza-associated mortality burden was the most frequently reported outcomes, while there was a dearth of measure of influenza-associated morbidity burden which may also bring considerable health impact and consequent economic impact. The challenge in estimating influenza-associated morbidity burden lies in the fact that compared with standardized death registry procedure in mainland China, hospital discharge data and outpatient data with well-defined population denominators were not readily available. Promoting standardization of electronic medical record system in public hospitals in China is a prerequisite for healthcare data analysis and sharing^61^. Studies in our review mainly located in more developed eastern provinces with a high number of scientific research institutes and scientists. Promoting interprovincial collaboration on scientific research and data sharing is crucial to strength influenza-associated morbidity estimates in central and western regions of China, with the ultimate goal to get nationally representative estimates of the full disease burden of influenza. The highest influenza-associated mortality in adults aged ≥ 65 years and morbidity in children aged < 5 years indicates that increasing influenza vaccination coverage in these two age groups is necessary. Urbanization was found positively correlated with influenza-associated ILI outpatient visits but was not correlated with influenza-associated deaths. Considering the government may not be able to provide free shots to every eligible individual because of huge fiscal expenditure, roll-out of vaccine campaign for children and older adults in urban areas with high population density first may prevent substantial outpatient visits thus reducing healthcare usage. Further studies of the relative burden of influenza in urban and rural areas would be worthwhile to guide the formulation of more refined or localized public health policies.

## Conclusion

In conclusion, more studies in less-developed central and western provinces are needed in the future to assess the geographical distribution patterns of influenza in China as a whole. People aged ≥ 65 years and < 5 years contribute mostly to mortality and morbidity burden due to influenza, which calls for targeted vaccination policy for older adults and younger children in mainland China.

## Supplementary Information

Supplementary Information.
