# Supplementary material for: Influenza-associated disease burden in mainland China: a systematic review and meta-analysis
Source: Sci Rep. 2021 Feb 3;11:2886. doi: 10.1038/s41598-021-82161-z (PMC7859194; doi:10.1038/s41598-021-82161-z)
Supplement: Supplementary file 1 — Supplementary Information. [file 41598_2021_82161_MOESM1_ESM.pdf]

Supplementary appendix

Influenza-associated disease burden in mainland China: a systematic review and meta-analysis

Jing Li, Yinzi Chen, Xiling Wang, Hongjie Yu

Table S1. Literature search strategy and results, by database.

Database	Search Strategy	Results
**PubMed**	1. influenza[all] OR flu[all]<br>2. burden[all] OR impact[all] OR incidence[all] OR morbidity[all] OR mortality[all] OR death[all] OR hospitalization[all] OR outpatient[all]<br>3. excess[all] OR influenza-associated[all] OR influenza-related[all] OR influenza-attributed[all] OR influenza-attributable[all] OR associated with influenza[all] OR related to influenza[all] OR attributed to influenza[all] OR attributable to influenza[all] OR flu-associated[all] OR flu-related[all] OR associated with flu[all] OR related to flu[all] OR attributed to flu[all] OR attributable to flu[all] OR confirmed influenza[all] OR confirmed flu[all]<br>4. China[all] OR Chinese[all]<br>5. avian[Title/Abstract] OR swine[Title/Abstract] OR zoonotic[title/abstract] OR h5n1[Title/Abstract] OR h5n2[Title/Abstract] OR h7n1[Title/Abstract] OR h7n2[Title/Abstract] OR h7n3[Title/Abstract] OR h7n7[Title/Abstract] OR h7n9[Title/Abstract] OR h9n2[Title/Abstract] OR h10n7[Title/Abstract] OR h10n8[Title/Abstract]<br>6. 1 AND 2 AND 3 AND 4 NOT 5<br>Filters: Publication date from 2005/01/01 to 2019/12/31; English	1367
**EMBASE**	1. (influenza OR flu).af.<br>2. (burden OR impact OR incidence OR morbidity OR mortality OR death OR hospitalization OR outpatient).af.<br>3. (excess OR influenza-associated OR influenza-related OR influenza-attributed OR influenza-attributable OR associated with influenza OR related to influenza OR attributed to influenza OR attributable to influenza OR flu-associated OR flu-related OR associated with flu OR related to flu OR attributed to flu OR attributable to flu OR confirmed influenza OR confirmed flu).af.<br>4. (China OR Chinese).af.<br>5. (avian OR swine OR zoonotic OR h5n1 OR h5n2 OR h7n1 OR h7n2 OR h7n3 OR h7n7 OR h7n9 OR h9n2 OR h10n7 OR h10n8).ti,ab.<br>6. 1 AND 2 AND 3 AND 4 NOT 5<br>limit 6 to (English language and yr="2005 - 2019")	218
**Web of Science**	1. TS = (influenza OR flu)<br>2. TS = (burden OR impact OR incidence OR morbidity OR mortality OR death OR hospitalization OR outpatient)<br>3. TS = (excess OR influenza-associated OR influenza-related OR	566

	influenza-attributed OR influenza-attributable OR associated with influenza OR related to influenza OR attributed to influenza OR attributable to influenza OR flu-associated OR flu-related OR associated with flu OR related to flu OR attributed to flu OR attributable to flu OR confirmed influenza OR confirmed flu)<br>4. TS = (China OR Chinese)<br>5. TS = (avian OR swine OR zoonotic OR h5n1 OR h5n2 OR h7n1 OR h7n2 OR h7n3 OR h7n7 OR h7n9 OR h9n2 OR h10n7 OR h10n8)<br>6. 1 AND 2 AND 3 AND 4 NOT 5<br>Time span = 2005-2019<br>Language = English	
**CNKI**	(SU = 流感 + 流行性感冒) AND (SU = 负担 + 影响 + 发病 + 死亡 + 住院 + 门诊) AND (FT = 超额 + 流感相关 + 流感导致 + 流感确诊) NOT (SU = 禽流感 + 猪流感)<br>年 between (2005, 2019)	418
**Wan-Fang Data**	(主题:(流感 OR 流行性感冒)*主题:(负担 OR 影响 OR 发病 OR 死亡 OR 住院 OR 门诊)*全部:(超额 OR 流感相关 OR 流感导致 OR 流感确诊)^主题:(禽流感 OR 猪流感))*Date:2005-2019	2814
**Chong-Qing VIP**	U = (流感 OR 流行性感冒) AND U = (负担 OR 影响 OR 发病 OR 死亡 OR 住院 OR 门诊) AND U = (超额 OR 流感相关 OR 流感导致 OR 流感确诊) NOT M = (禽流感 OR 猪流感) AND (years: [2005 TO 2019])	36
**CBM**	((("流感"[常用字段:智能] OR "流行性感冒"[常用字段:智能]) AND("负担"[常用字段:智能] OR "影响"[常用字段:智能] OR "发病"[常用字段:智能] OR "死亡"[常用字段:智能] OR "住院"[常用字段:智能] OR "门诊"[常用字段:智能]) AND("超额"[常用字段:智能] OR "流感相关"[常用字段:智能] OR "流感导致"[常用字段:智能] OR "流感确诊"[常用字段:智能])) NOT("禽流感"[常用字段:智能] OR "猪流感"[常用字段:智能])) AND 2005-2019[日期]	296

Table S2. Quality assessment checklist for modeling studies.

Checklist item	Yes	No	Unclear	Not applicable
1. Was there clear statement about the questions that the model aimed to answer?				
2. Were the sources of data clearly described?				
3. Was there clear statement about what model was used?				
4. Was/Were the model structure or variables used in the model clearly described?				
5. Were necessary estimates (e.g. burden measure) available?				
6. Were standard errors or 95% confidence intervals of the estimates available?				
7. Was model fit information (e.g. $\boldsymbol{R^2}$) available?				
8. Was model validation performed?				
9. Were sensitivity analyses carried out?				
10. Was there explanation about how missing data were addressed?				

Note: Yes (score=1), No (score=0), Unclear (score=0), Not applicable (score=1).

Table S3. Quality assessment checklist for laboratory-confirmed studies.

Checklist item	Yes	No	Unclear	Not applicable
1. Were there clear criteria for inclusion in the case series?				
2. Was the condition measured in a standard, reliable way for all participants included in the case series?				
3. Were valid methods used for identification of the condition for all participants included in the case series?				
4. Did the case series have consecutive inclusion of participants?				
5. Did the case series have complete inclusion of participants?				
6. Was there clear reporting of the demographics of the participants in the study?				
7. Was there clear reporting of clinical information of the participants?				
8. Were the outcomes or follow up results of cases clearly reported?				
9. Was there clear reporting of the presenting site(s)/clinic(s) demographic information?				
10. Was statistical analysis appropriate?				

Note: Yes (score=1), No (score=0), Unclear (score=0), Not applicable (score=1)

Table S4. Characteristics of included studies about influenza-associated disease burden.

Characteristics	No. of studies	
	Modeling approach (n=22*)	Laboratory-confirmed approach (n=8)
Type of disease burden		
Mortality burden	17	0
Hospitalization burden	2	6
Outpatient burden	3	2
Statistical model		
Multiplier method	2	-
Serfling model	5	-
Rate difference model	2	-
Regression model with an influenza activity proxy	18	-
Proxy used for influenza activity		
LAB number	1	-
LAB%	14	-
ILI%	1	-
LAB%×ILI%	3	-
Covariates considered in the regression models		
Smoothing function of time	7	-
Polynomial function of time	15	-
Absolute humidity	2	-
Temperature + absolute humidity	3	-
Temperature + relative humidity	2	-
Diagnostic test		
RT-PCR	-	6
IFA	-	1
HI	-	1
Case definition		
SARI	-	5
Pneumonia	-	1
ILI	-	2
Geographic location		
Northern China	35	2
Southern China	41	6
Study period		
Pre-pandemic	7	2
Post-pandemic	11	6
Both	4	0

LAB number, positive number of laboratory-confirmed influenza; LAB%, positive proportion of laboratory-confirmed influenza; ILI%, influenza-like illness consultation rate; LAB%×ILI%, product of positive

proportion of laboratory-confirmed influenza and influenza-like illness consultation rate. RT-PCR, reverse transcription-polymerase chain reaction; IFA, immunofluorescence assay; HI, hemagglutination inhibition assay. SARI, severe acute respiratory infection; ILI, influenza-like illness.

* The subtotal in each category may differ from the total number of modeling studies (n=22) because some studies could have estimated influenza disease burden using multiple statistical models or influenza activity proxies or for multiple provinces or cities.

Table S5. Basic information on included modeling studies.

Reference	Language	Region	Year / Influenza season	Predominant virus*	Statistical model	Influenza activity proxy	Outcome measurement	Age group
Influenza-associated mortality burden								
Chen, 2010[1]	Chinese	Qingdao	2005/06-2007/08[a]	Not reported	Serfling	N	AC, R&C	all ages, ≥65 y, <65 y
Yang, 2011[2]	English	Guangzhou	2005-06[a]	2005 A(H3N2), 2006 A(H1N1)	Poisson	LAB%	AC	all ages
Feng, 2012[3]	English	3 northern cities and 5 southern cities[c]	2005/06-2008/09[a]	2005/06 A(H1N1), 2006/07 A(H3N2), 2007/08 B, 2008/09 A(H1N1)	Serfling, NB	N, LAB%	AC, R&C, P&I, COPD, IHD	all ages, ≥65 y, <65 y
Yu, 2013[4]	English	Rural and urban DSP sites of northern and southern China	2005/06-2008/09[ab]	2005/06 A(H1N1), 2006/07 A(H3N2), 2007/08 B, 2008/09 A(H1N1)	NB	LAB%	AC, R&C, Res	all ages, ≥65 y, <65 y
Yang, 2013[5]	Chinese	Harbin	2005-08[b]	Not reported	RD, Poisson	N, Unclear	AC, R&C, P&I, COPD, IHD	all ages, ≥65 y
Wang, 2014[6]	English	Guangzhou	2010-12	2010 NA, 2011 A(H1N1)pdm09, 2012 A(H3N2)	NB	LAB%	AC, R&C, P&I, COPD, IHD	all ages, ≥65 y, <65 y
Li, 2015[7]	Chinese	Dalian	2005/06-2007/08[a]	Not reported	Serfling, NB	N, LAB%	AC, R&C	all ages, ≥65 y
Guo, 2016[8]	English	Zhuhai	2007-08	2007 A(H3N2), 2008 NA	Poisson	ILI%	Res, COPD	all ages, ≥65 y, <65 y
Zhang, 2016[9]	Chinese	Shunyi (Beijing)	2010/11-2014/15	Not reported	NB	LAB%	AC, R&C, P&I	all ages, ≥65 y, <65 y

Lao, 2016[10]	Chinese	Ningbo	2010-14	2010 B, 2011 A(H1N1)pdm09, 2012 B, 2013 A(H3N2), 2014 NA	RD	N	AC, R&C, P&I, COPD, IHD	all ages, ≥65 y
Liu, 2017[11]	English	Hefei	2010-15	2010 B, 2011 B, 2012 A(H3N2), 2013 A(H3N2), 2014 A(H3N2), 2015 A(H3N2)	Poisson	LAB%×ILI%	AC, Res, P&I, COPD, IHD	all ages
Huang, 2017[12]	Chinese	Shenzhen	2013-15	Not reported	Serfling	N	AC, R&C, P&I	all ages, ≥65 y, <65 y
Yu, 2017[13]	English	Shanghai	2010-15	2010 B, 2011 B, 2012 A(H3N2), 2013 A(H3N2), 2014 A(H3N2), 2015 A(H3N2)	Poisson	LAB number, LAB%, LAB%×ILI%	P&I	all ages, ≥60 y, <60 y
Zhang, 2018[14]	English	Yancheng	2011-15	Not reported	Linear	LAB%×ILI%	AC, R&C, Res	all ages, ≥65 y
Wu, 2018[15]	English	Beijing	2007/08-2012/13[b]	Not reported	NB	LAB%	AC, R&C	all ages, ≥65 y, <65 y
Li, 2019[16]	English	22 provinces[d]	2010/11-2014/15	Not reported	Linear	LAB%	Res	all ages, ≥60 y, <60 y
Zhao, 2019[17]	Chinese	Shanxi	2013/14-2016/17	2013/14 NA, 2014/15 A(H3N2), 2015/16 B, 2016/17 A(H3N2)	Serfling, Linear, Poisson	N, LAB%, LAB%	AC, R&C, Res	all ages
Influenza-associated hospitalization burden								
Feng, 2014[18]	Chinese	Wuxi	2005-10[b]	Not reported	NB	LAB%	Res, P&I	<15 y
Zhang, 2017[19]	English	Suzhou	2005/06-2010/11[b]	2005/06 A(H1N1), 2006/07 A(H3N2), 2007/08 NA, 2008/09 A(H3N2), 2010/11 NA	NB	LAB%	P&I	<5 y
Influenza-associated outpatient burden								

Wu, 2017[20]	English	Beijing	2015/16	Not reported	Multiplier	N	ILI	all ages, 0-4 y, 5-14 y, 15-24 y, 25-59 y, ≥60 y
Zhang, 2019[21]	Chinese	Beijing	2017/18	Not reported	Multiplier	N	ILI	all ages, 0-4 y, 5-14 y, 15-24 y, 25-59 y, ≥60 y
Feng, 2019[22]	English	30 provinces[e]	2006-15[b]	Not reported	Linear	LAB%	ILI	all ages, 0-14 y, 15-59 y, ≥60 y

NA, no predominant virus. NB, negative binomial model; RD, rate difference model. LAB number, the positive numbers of laboratory-confirmed influenza; LAB%, the positive proportions of laboratory-confirmed influenza; ILI%, influenza-like illness consultation rate; LAB%×ILI%, product of positive proportions of laboratory-confirmed influenza and influenza-like illness consultation rate. N, no influenza activity proxy used in the model. AC, all-cause; R&C, respiratory and circulatory disease; Res, respiratory disease; P&I, pneumonia and influenza; COPD, chronic obstructive pulmonary disease; IHD, ischemic heart disease; ILI, influenza-like illness.

* An influenza type or subtype was considered as predominant during an influenza season when it accounted for at least 50% of the specimens tested positive for influenza.

[a] Years before 2005 were excluded.

[b] Pandemic period was excluded.

[c] 3 northern cities included Dalian, Qingdao, and Zhaoyuan (Yantai); 5 southern cities included Shanghai, Wuhan, Yichang, Ningbo, and Guangzhou.

[d] 22 provinces included 9 northern provinces (Heilongjiang, Liaoning, Jilin, Beijing, Tianjin, Gansu, Shandong, Shaanxi, and Henan) and 13 southern provinces (Jiangsu, Anhui, Shanghai, Hubei, Sichuan, Zhejiang, Chongqing, Jiangxi, Hunan, Guizhou, Fujian, Guangxi, and Guangdong).

[e] 30 provinces included 15 northern provinces (Heilongjiang, Xinjiang, Inner Mongolia, Liaoning, Jilin, Beijing, Tianjin, Hebei, Ningxia, Shanxi, Gansu, Shandong, Qinghai, Shaanxi, and Henan) and 15 southern provinces (Jiangsu, Anhui, Shanghai, Hubei, Sichuan, Zhejiang, Chongqing, Jiangxi, Hunan, Guizhou, Fujian, Yunnan, Guangxi, Guangdong, and Hainan).

Table S6. Basic information on included laboratory-confirmed studies.

Reference	Language	Region	Year / Influenza season	Predominant virus*	Specimen type	Diagnostic test	Outcome measurement	Case definition	Age group
Influenza-associated hospitalization burden									
Ji, 2010[23]	English	Suzhou	2007-08	A	Nasal aspirates	IFA	Pneumonia	Clinical diagnosis	<5 y
Yu, 2014[24]	English	Jingzhou	2010/11-2011/12	Not reported	NP swabs	RT-PCR	SARI	An elevated temperature (rectal or axillary temperature ≥37.3 °C) and at least one sign or symptom of acute respiratory illness, including cough, sore throat, tachypnea, difficulty breathing, abnormal breath sounds on auscultation, sputum production, hemoptysis, chest pain, or chest radiograph consistent with pneumonia.	all ages, 0-4 y, 5-14 y, 15-64 y, ≥65 y
Zhao, 2018[25]	Chinese	Huairou (Beijing)	2017/18	NA	Throat swabs	RT-PCR	SARI	An acute respiratory infection with: 1) history of fever or measure fever of ≥38 °C; 2) and cough; 3) with onset within the last 10 days; 4) and requires hospitalization.	all ages, 0-4 y, 5-14 y, 15-64 y, ≥65 y
Zhang, 2018[26]	English	Beijing	2014/15-2015/16	Not reported	Throat swabs	RT-PCR	SARI	An acute respiratory infection with: 1) history of fever or measure fever of ≥38 °C; 2) and cough; 3) with onset	all ages, 0-4 y, 5-14 y, 15-59 y, ≥60 y

								within the last 10 days; 4) and requires hospitalization.	
Yu, 2019[27]	English	Suzhou	2011/12-2015/16	2011/12 A(H3N2), 2012/13 NA, 2013/14 A(H3N2), 2014/15 A(H3N2), 2015/16 A(H1N1)pdm09 and B	NP aspirates	RT-PCR	SARI	The presence of fever (measured axillary temperature ≥38 °C) and cough or sore/inflamed or red pharynx in the 7 days preceding hospitalization.	<5 y
Zhang, 2019[28]	Chinese	Suzhou	2016/17-2017/18	2016/17 A(H3N2), 2017/18 A(H1N1)pdm09	NP aspirates	RT-PCR	SARI	The presence of fever (measured axillary temperature ≥38 °C) and cough or sore/inflamed or red pharynx in the 7 days preceding hospitalization.	<5 y
Influenza-associated outpatient burden									
Guo, 2012[29]	English	Zhuhai	2008[a]	NA	Throat swabs	HI	ILI	Sudden onset of fever (38 °C or above) with cough, sore throat, or other respiratory symptoms.	all ages, 0-4 y, 5-14 y, 15-24 y, 25-59 y, ≥60 y
Gao, 2019[30]	Chinese	Suzhou	2011/12-2016/17	2011/12 B, 2012/13 NA, 2013/14 A(H3N2), 2014/15 B, 2015/16 A(H1N1)pdm09, 2016/17 A(H3N2)	Throat swabs	RT-PCR	ILI	Fever (axillary temperature ≥38 °C), with cough or sore throat, and in the absence of other laboratory confirmed evidence.	<5 y

NA, no predominant virus. NP, nasopharyngeal. IFA, immunofluorescence assay; RT-PCR, reverse transcription polymerase chain reaction; HI, hemagglutination inhibition assay. SARI, severe acute respiratory infection; ILI, influenza-like illness.

* An influenza type or subtype was considered as predominant during an influenza season when it accounted for at least 50% of the specimens tested positive for influenza.

[a] Pandemic period was excluded.

Table S7. Quality assessment for included modeling studies.

Reference	1. Was there clear statement about the questions that the model aimed to answer?	2. Were the sources of data clearly described?	3. Was there clear statement about what model was used?	4. Was/Were the model structure or variables used in the model clearly described?	5. Were necessary estimates (e.g. burden measure) available?	6. Were standard errors or 95% confidence intervals of the estimates available?	7. Was model fit information (e.g. R^2) available?	8. Was model validation perform?	9. Were sensitivity analyses carried out?	10. Was there explanation about how missing data were addressed?	Score obtained
Chen, 2010[1]	Y	Y	Y	Y	Y	Y	Y	NA	N	Unclear	8
Yang, 2011[2]	Y	Y	Y	Y	Y	Y	N	NA	N	Unclear	7
Feng, 2012[3]	Y	Y	Y	Y	Y	Y	Y	NA	Y	Unclear	9
Yu, 2013[4]	Y	Y	Y	Y	Y	Y	Y	NA	Y	Unclear	9
Yang, 2013[5]	Y	Y	Y	Y	Y	N	NA	NA	N	Unclear	7
Wang, 2014[6]	Y	Y	Y	Y	Y	Y	N	NA	Y	Unclear	8
Li, 2015[7]	Y	Y	Y	Y	Y	Y	Y	NA	Y	Unclear	9
Guo, 2016[8]	Y	Y	Y	Y	Y	N	Y	NA	Y	Unclear	8
Zhang,	Y	Y	Y	Y	Y	Y	Y	NA	Y	Unclear	9

2016[9]											
Lao, 2016[10]	Y	Y	Y	Y	Y	N	NA	NA	N	Unclear	7
Liu, 2017[11]	Y	Y	Y	Y	Y	Y	N	NA	Y	Unclear	8
Huang, 2017[12]	Y	Y	Y	Y	Y	Y	Y	NA	N	Unclear	8
Yu, 2017[13]	Y	Y	Y	Y	Y	Y	Y	NA	Y	Unclear	9
Zhang, 2018[14]	Y	Y	Y	Y	Y	Y	N	NA	Y	Unclear	8
Wu, 2018[15]	Y	Y	Y	Y	Y	Y	Y	NA	N	Unclear	8
Li, 2019[16]	Y	Y	Y	Y	Y	Y	N	NA	Y	Unclear	8
Zhao, 2019[17]	Y	Y	Y	Y	Y	N	Y	NA	Y	Unclear	8
Feng, 2014[18]	Y	Y	Y	Y	Y	Y	Y	N	N	Unclear	7
Zhang, 2017[19]	Y	Y	Y	Y	Y	Y	Y	N	N	Y	8
Wu, 2017[20]	Y	Y	Y	Y	Y	Y	NA	N	Y	Unclear	8
Zhang, 2019[21]	Y	Y	Y	Y	Y	Y	NA	N	N	Unclear	7
Feng, 2019[22]	Y	Y	Y	Y	Y	Y	N	N	Y	Y	8

Y, yes; N, no; NA, not applicable.

Table S8. Quality assessment for included laboratory-confirmed studies.

Reference	1. Were there clear criteria for inclusion in the case series?	2. Was the condition measured in a standard, reliable way for all participants included in the case series?	3. Were valid methods used for identification of the condition for all participants included in the case series?	4. Did the case series have consecutive inclusion of participants?	5. Did the case series have complete inclusion of participants?	6. Was there clear reporting of the demographics of the participants in the study?	7. Was there clear reporting of clinical information of the participants?	8. Were the outcomes or follow up results of cases clearly reported?	9. Was there clear reporting of the presenting site(s)/clinic(s) demographic information?	10. Was statistical analysis appropriate?	Score obtained
Ji, 2010[23]	Y	Y	Y	Y	Y	Y	Y	N	Y	Y	9
Yu, 2014[24]	Y	Y	Y	Y	Y	Y	Y	Y	Y	Y	10
Zhao, 2018[25]	Y	Y	Y	Y	Y	Y	Y	N	Y	Y	9
Zhang, 2018[26]	Y	Y	Y	Y	Y	Y	Y	Y	Y	Y	10
Yu, 2019[27]	Y	Y	Y	Y	Y	Y	Y	Y	Y	Y	10
Zhang, 2019[28]	Y	Y	Y	Y	Y	Y	Y	N	Y	Y	9
Guo, 2012[29]	Y	Y	Y	Y	Y	Y	Y	N	Y	Y	9
Gao, 2019[30]	Y	Y	Y	Y	Y	Y	Y	N	Y	Y	9

Y, yes; N, no; NA, not applicable.

Table S9. Subgroup analyses for the estimates of influenza-associated mortality.

Variable	No. of estimates (n=245)	Influenza-associated mortality rates (95% CI)	I^2(%)	*P* value[a]	*P* value[b]
Age group					
All ages	102	7.56 (6.34, 8.79)	98.6	<0.001	<0.001
<65 years	62	1.34 (0.97, 1.70)	85.8	<0.001	
≥65 years	81	62.04 (50.08, 74.01)	99.8	<0.001	
Cause of death					
All-cause	52	45.67 (28.35, 63.00)	99.7	<0.001	<0.001
R&C	48	35.39 (21.34, 49.43)	99.7	<0.001	
Res	87	16.45 (11.51, 21.39)	99.1	<0.001	
P&I	26	3.53 (0.55, 6.50)	96.5	<0.001	
COPD	16	7.64 (1.81, 13.47)	98.5	<0.001	
IHD	16	6.96 (1.79, 12.12)	98.9	<0.001	
Statistical model					
Serfling model	35	19.24 (6.35, 32.13)	89.6	<0.001	0.852
Rate difference model	15	30.99 (-0.97, 62.94)	99.7	<0.001	
Regression model with an influenza activity proxy	195	23.38 (17.84, 28.92)	99.6	<0.001	
Geographic location					
Northern China	118	24.65 (17.07, 32.23)	99.6	<0.001	0.426
Southern China	127	22.06 (15.20, 28.91)	99.6	<0.001	
Study period					
Pre-pandemic	104	28.98 (19.84, 38.12)	99.7	<0.001	0.221
Post-pandemic	135	18.12 (12.53, 23.71)	99.3	<0.001	
Both	6	38.16 (-10.01, 86.33)	99.8	<0.001	

R&C, respiratory and circulatory disease; Res, respiratory disease; P&I, pneumonia and influenza; COPD, chronic obstructive pulmonary disease; IHD, ischemic heart disease.

[a] *P* value for heterogeneity within each subgroup.

[b] *P* value for heterogeneity between subgroups.

Table S10. Pooled influenza-associated mortality rates per 100,000 persons by cause of death and age group in sensitivity analyses.

Outcome	No. of estimates	Influenza-associated mortality rates (95% CI)
All-cause		
All ages	19	13.38 (10.35, 16.41)
Age-standardized	12	14.76 (11.54, 17.98)
<65 years	12	2.67 (2.27, 3.07)
≥65 years	15	114.43 (82.02, 146.84)
Respiratory and circulatory disease		
All ages	18	10.43 (8.12, 12.74)
Age-standardized	12	11.01 (8.77, 13.26)
<65 years	12	1.55 (1.30, 1.80)
≥65 years	15	92.19 (67.63, 116.76)
Respiratory disease		
All ages	32	5.84 (4.66, 7.03)
Age-standardized	27	5.03 (3.91, 6.16)
<65 years	27	1.23 (0.64, 1.82)
≥65 years	28	43.71 (34.33, 53.09)
Pneumonia and influenza*		
All ages	10	0.63 (0.18, 1.08)
≥65 years	9	7.69 (-0.59, 15.96)
Chronic obstructive pulmonary disease*		
All ages	6	2.88 (1.41, 4.35)
Ischemic heart disease*		
All ages	5	2.11 (0.96, 3.26)

* Due to the small numbers (<5) of the reported estimates for age-standardized, <65 years, and ≥65 years, the meta-analyses were not performed.

Table S11. Estimates of laboratory-confirmed influenza hospitalization by age group.

Study	Region	SARI/ Pneumonia	Confirmed influenza cases	Catchment population	Rate per 100,000 persons (95 % CI)
All ages					
Yu, 2013	Jingzhou	SARI	982	763428	129 (127, 131)
Zhao, 2018	Huairou (Beijing)	SARI	148	260726	55 (47, 64)
Zhang, 2018	Beijing	SARI	321	842895	38 (34, 43)
0-4 years					
Ji, 2010	Suzhou	Pneumonia	60	240735	25 (19, 31)
Yu, 2013	Jingzhou	SARI	673	30786	2185 (2159, 2212)
Zhao, 2018	Huairou (Beijing)	SARI	65	13095	499 (353, 702)
Zhang, 2018	Beijing	SARI	155	34948	442 (367, 506)
Yu, 2019	Suzhou	SARI	836	128135	715 (569, 861)
Zhang, 2019	Suzhou	SARI	1559	226539	688 (656, 720)
5-14 years					
Yu, 2013	Jingzhou	SARI	172	45254	382 (356, 417)
Zhao, 2018	Huairou (Beijing)	SARI	17	22270	76 (47, 122)
Zhang, 2018	Beijing	SARI	68	62100	109 (85, 138)
15-64 years					
Yu, 2013	Jingzhou	SARI	84	440502	19 (17, 19)
Zhao, 2018	Huairou (Beijing)	SARI	35	209337	17 (11, 26)
Zhang, 2018*	Beijing	SARI	34	658386	5 (3, 8)
≥65 years					
Yu, 2013	Jingzhou	SARI	54	47108	115 (110, 117)
Zhao, 2018	Huairou (Beijing)	SARI	30	24843	123 (86, 175)
Zhang, 2018†	Beijing	SARI	75	87461	86 (68, 108)

SARI, severe acute respiratory infection.

* Age 15-59 years

† Age ≥60 year

A. All ages

Study	Region	Model	EMR	95% CI
Yang, 2013	Harbin	RD	25.59	(22.89, 28.29)
Yang, 2013	Harbin	Poisson	18.83	(16.32, 21.34)
Li, 2015	Dalian	Serfling	3.97	(1.50, 6.47)
Li, 2015	Dalian	NB	6.17	(1.70, 38.27)
Zhang, 2016	Shunyi (Beijing)	NB	8.80	(3.40, 15.10)
Wu, 2018	Beijing	NB	17.20	(7.20, 67.50)
Zhao, 2019	Shanxi	Serfling	6.47	(4.31, 8.63)
Zhao, 2019	Shanxi	Linear	21.30	(18.72, 23.88)
Zhao, 2019	Shanxi	Poisson	21.76	(19.17, 24.35)
Chen, 2010	Qingdao	Serfling	18.30	(8.57, 27.97)
Feng, 2012	Northern cities	Serfling	14.97	(6.67, 24.33)
Feng, 2012	Northern cities	NB	21.10	(8.33, 74.90)
Yu, 2013	Northern rural sities	NB	17.88	(9.68, 59.85)
Yu, 2013	Northern urban sities	NB	19.48	(10.18, 56.08)
Zhang, 2018	Yancheng	Linear	6.94	(2.85, 14.28)
Liu, 2017	Hefei	Poisson	9.90	(6.28, 13.52)
Lao, 2016	Ningbo	RD	18.74	(16.24, 21.24)
Yang, 2011	Guangzhou	Poisson	11.55	(-2.00, 20.20)
Wang, 2014	Guangzhou	NB	14.72	(12.12, 17.31)
Huang, 2017	Shenzhen	Serfling	6.11	(2.49, 9.73)
Feng, 2012	Southern cities	NB	12.47	(1.83, 51.67)
Yu, 2013	Southern rural sities	NB	8.10	(0.73, 61.00)
Yu, 2013	Southern urban sities	NB	20.88	(9.83, 56.85)
**Overall (I^2=95.9%, p<0.001)**			**14.33**	**(11.56, 17.10)**

Note: Random-effects model

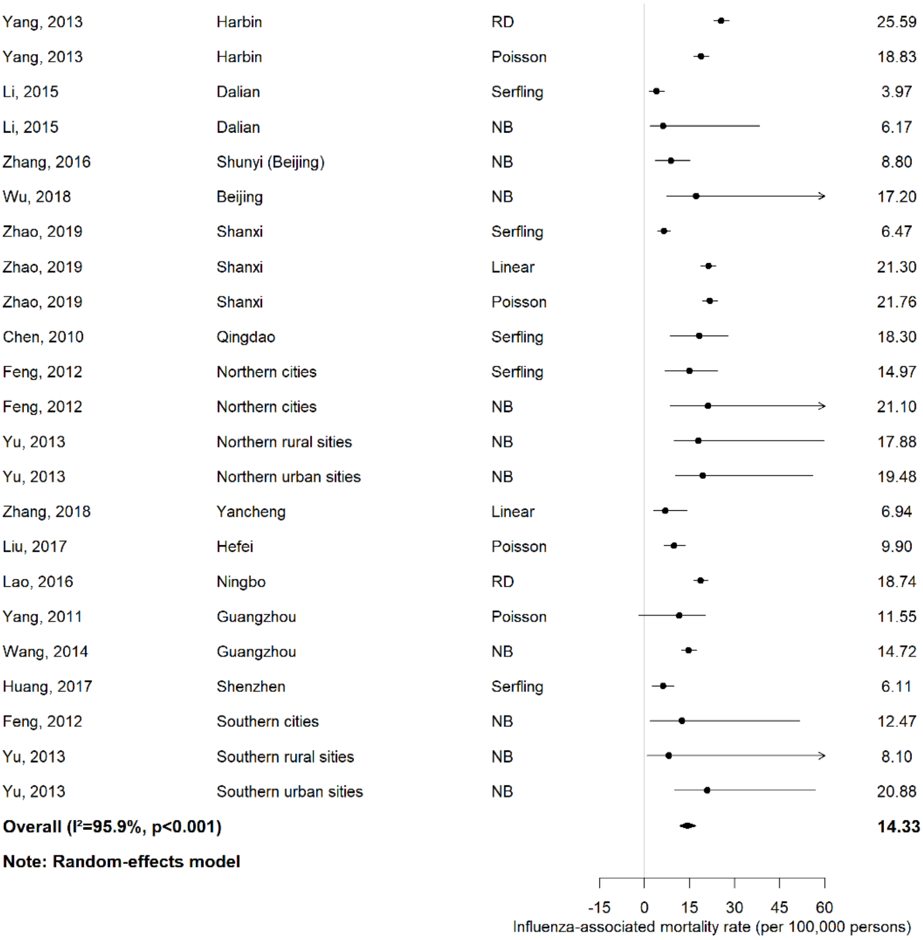

B. Age-standardized

Study	Region	Model	EMR	95% CI
Zhang, 2016	Shunyi (Beijing)	NB	8.19	(3.14, 14.19)
Wu, 2018	Beijing	NB	12.55	(5.00, 47.74)
Chen, 2010	Qingdao	Serfling	12.96	(5.90, 19.98)
Feng, 2012	Northern cities	Serfling	12.22	(5.11, 20.25)
Feng, 2012	Northern cities	NB	16.74	(6.49, 62.78)
Yu, 2013	Northern rural sities	NB	21.28	(11.71, 69.89)
Yu, 2013	Northern urban sities	NB	18.04	(9.39, 52.22)
Wang, 2014	Guangzhou	NB	18.83	(15.43, 22.23)
Huang, 2017	Shenzhen	Serfling	23.66	(8.21, 39.11)
Feng, 2012	Southern cities	NB	9.15	(1.27, 39.30)
Yu, 2013	Southern rural sities	NB	8.68	(0.77, 64.87)
Yu, 2013	Southern urban sities	NB	20.27	(9.50, 55.23)
**Overall (I^2=91.3%, p<0.001)**			**14.76**	**(11.54, 17.98)**

Note: Random-effects model

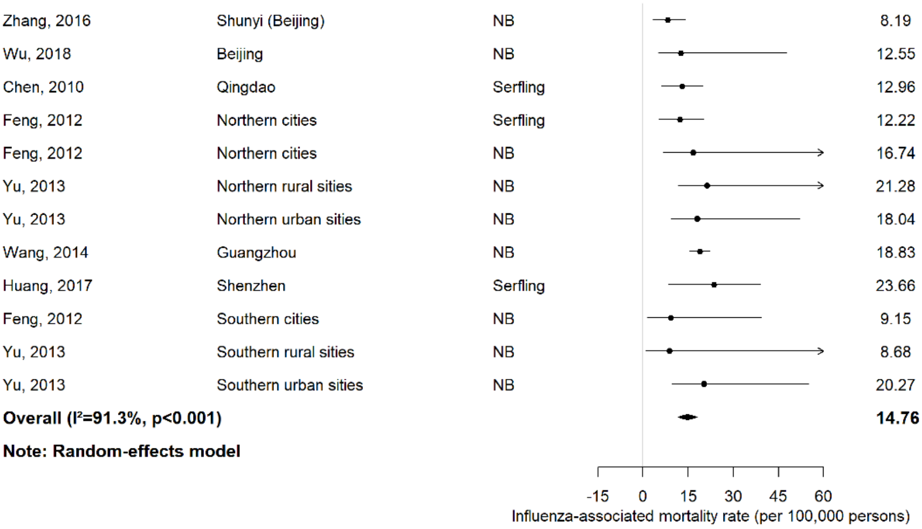

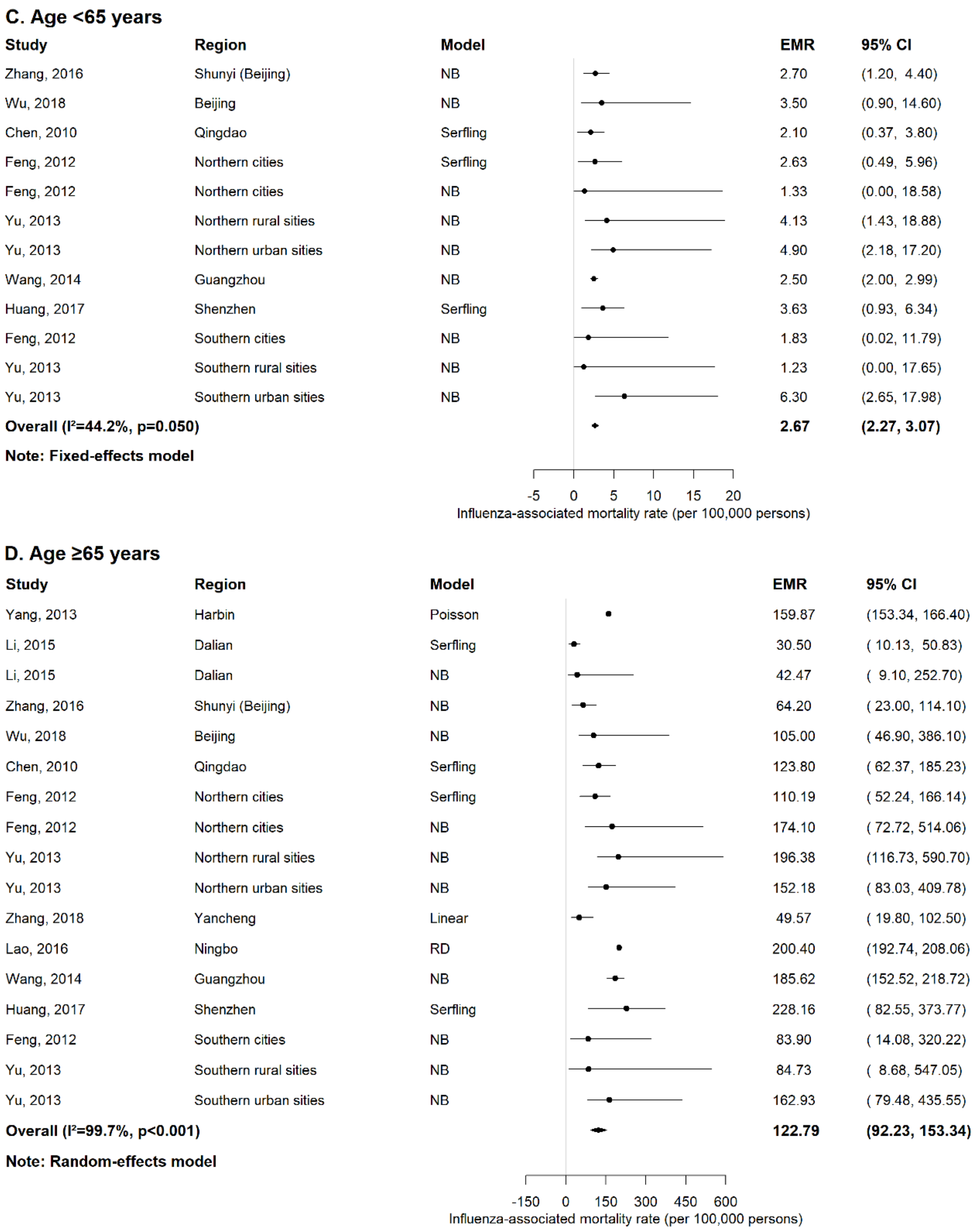

Figure S1. Age-specific influenza-associated all-cause mortality rates. EMR, excess mortality rate; RD, rate difference model; NB, negative binomial model.

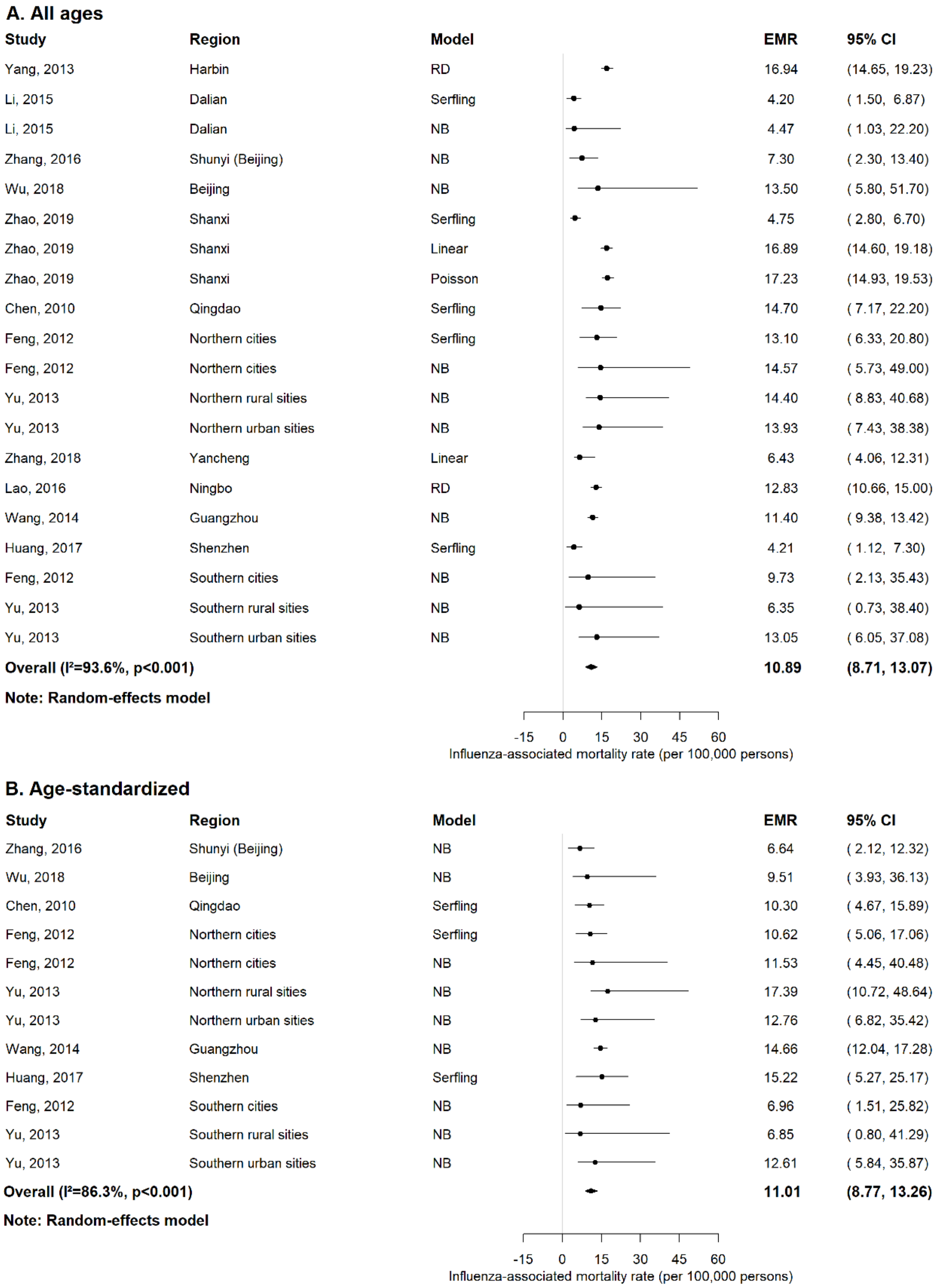
A. All ages
Study
Region
Model
EMR
95% CI
Yang, 2013 Harbin RD 16.94 (14.65, 19.23)
Li, 2015 Dalian Serfling 4.20 (1.50, 6.87)
Li, 2015 Dalian NB 4.47 (1.03, 22.20)
Zhang, 2016 Shunyi (Beijing) NB 7.30 (2.30, 13.40)
Wu, 2018 Beijing NB 13.50 (5.80, 51.70)
Zhao, 2019 Shanxi Serfling 4.75 (2.80, 6.70)
Zhao, 2019 Shanxi Linear 16.89 (14.60, 19.18)
Zhao, 2019 Shanxi Poisson 17.23 (14.93, 19.53)
Chen, 2010 Qingdao Serfling 14.70 (7.17, 22.20)
Feng, 2012 Northern cities Serfling 13.10 (6.33, 20.80)
Feng, 2012 Northern cities NB 14.57 (5.73, 49.00)
Yu, 2013 Northern rural sities NB 14.40 (8.83, 40.68)
Yu, 2013 Northern urban sities NB 13.93 (7.43, 38.38)
Zhang, 2018 Yancheng Linear 6.43 (4.06, 12.31)
Lao, 2016 Ningbo RD 12.83 (10.66, 15.00)
Wang, 2014 Guangzhou NB 11.40 (9.38, 13.42)
Huang, 2017 Shenzhen Serfling 4.21 (1.12, 7.30)
Feng, 2012 Southern cities NB 9.73 (2.13, 35.43)
Yu, 2013 Southern rural sities NB 6.35 (0.73, 38.40)
Yu, 2013 Southern urban sities NB 13.05 (6.05, 37.08)
Overall (I²=93.6%, p<0.001) 10.89 (8.71, 13.07)
Note: Random-effects model
-15 0 15 30 45 60
Influenza-associated mortality rate (per 100,000 persons)
B. Age-standardized
Study
Region
Model
EMR
95% CI
Zhang, 2016 Shunyi (Beijing) NB 6.64 (2.12, 12.32)
Wu, 2018 Beijing NB 9.51 (3.93, 36.13)
Chen, 2010 Qingdao Serfling 10.30 (4.67, 15.89)
Feng, 2012 Northern cities Serfling 10.62 (5.06, 17.06)
Feng, 2012 Northern cities NB 11.53 (4.45, 40.48)
Yu, 2013 Northern rural sities NB 17.39 (10.72, 48.64)
Yu, 2013 Northern urban sities NB 12.76 (6.82, 35.42)
Wang, 2014 Guangzhou NB 14.66 (12.04, 17.28)
Huang, 2017 Shenzhen Serfling 15.22 (5.27, 25.17)
Feng, 2012 Southern cities NB 6.96 (1.51, 25.82)
Yu, 2013 Southern rural sities NB 6.85 (0.80, 41.29)
Yu, 2013 Southern urban sities NB 12.61 (5.84, 35.87)
Overall (I²=86.3%, p<0.001) 11.01 (8.77, 13.26)
Note: Random-effects model
-15 0 15 30 45 60
Influenza-associated mortality rate (per 100,000 persons)

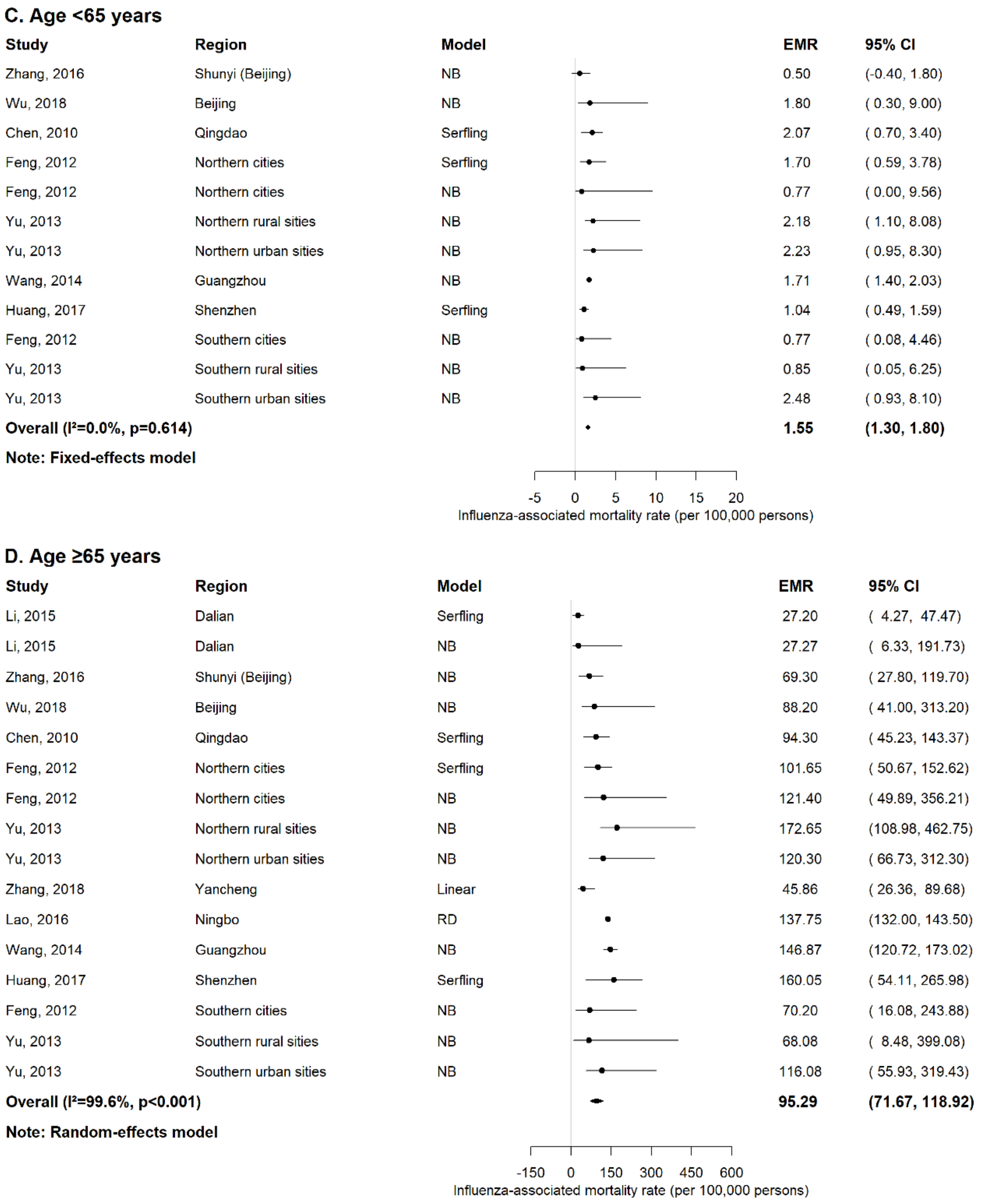

Figure S2. Age-specific influenza-associated R&C mortality rates. R&C, respiratory and circulatory; EMR, excess mortality rate; RD, rate difference model; NB, negative binomial model.

A. All ages

Study	Region	Model	EMR	95% CI
Li, 2018	Heilongjiang	Linear	3.00	(2.50, 3.40)
Li, 2018	Liaoning	Linear	4.20	(3.60, 5.20)
Li, 2018	Jilin	Linear	3.60	(3.10, 4.10)
Li, 2018	Beijing	Linear	12.60	(11.20, 14.00)
Li, 2018	Tianjin	Linear	3.70	(2.80, 4.50)
Zhao, 2019	Shanxi	Serfling	0.46	(-1.20, 2.12)
Zhao, 2019	Shanxi	Linear	2.41	(0.69, 4.13)
Zhao, 2019	Shanxi	Poisson	2.05	(0.34, 3.76)
Li, 2018	Gansu	Linear	12.00	(10.20, 14.00)
Li, 2018	Shandong	Linear	7.40	(6.70, 8.40)
Li, 2018	Shaanxi	Linear	6.20	(5.40, 6.90)
Li, 2018	Henan	Linear	2.70	(2.20, 3.30)
Yu, 2013	Northern rural sities	NB	5.40	(3.65, 13.50)
Yu, 2013	Northern urban sities	NB	4.43	(2.55, 12.30)
Zhang, 2018	Yancheng	Linear	4.59	(3.94, 7.41)
Li, 2018	Jiangsu	Linear	7.90	(6.90, 8.90)
Liu, 2017	Hefei	Poisson	2.74	(1.87, 3.50)
Li, 2018	Anhui	Linear	4.90	(4.10, 5.60)
Li, 2018	Shanghai	Linear	13.00	(11.20, 14.70)
Li, 2018	Hubei	Linear	3.70	(3.00, 4.40)
Li, 2018	Sichuan	Linear	5.10	(3.80, 7.00)
Li, 2018	Zhejiang	Linear	9.40	(8.70, 10.10)
Li, 2018	Chongqing	Linear	8.60	(6.60, 10.50)
Li, 2018	Jiangxi	Linear	1.70	(0.30, 3.20)
Li, 2018	Hunan	Linear	6.70	(5.70, 8.20)
Li, 2018	Guizhou	Linear	9.80	(7.40, 12.20)
Li, 2018	Fujian	Linear	8.10	(7.00, 9.30)
Li, 2018	Guangxi	Linear	11.00	(8.80, 13.00)
Guo, 2016	Zhuhai	Poisson	6.49	(4.66, 8.32)
Li, 2018	Guangdong	Linear	5.30	(3.70, 7.30)
Yu, 2013	Southern rural sities	NB	3.83	(0.98, 16.00)
Yu, 2013	Southern urban sities	NB	5.10	(2.38, 14.30)
**Overall (I²=96.4%, p<0.001)**			**5.84**	**(4.66, 7.03)**

Note: Random-effects model

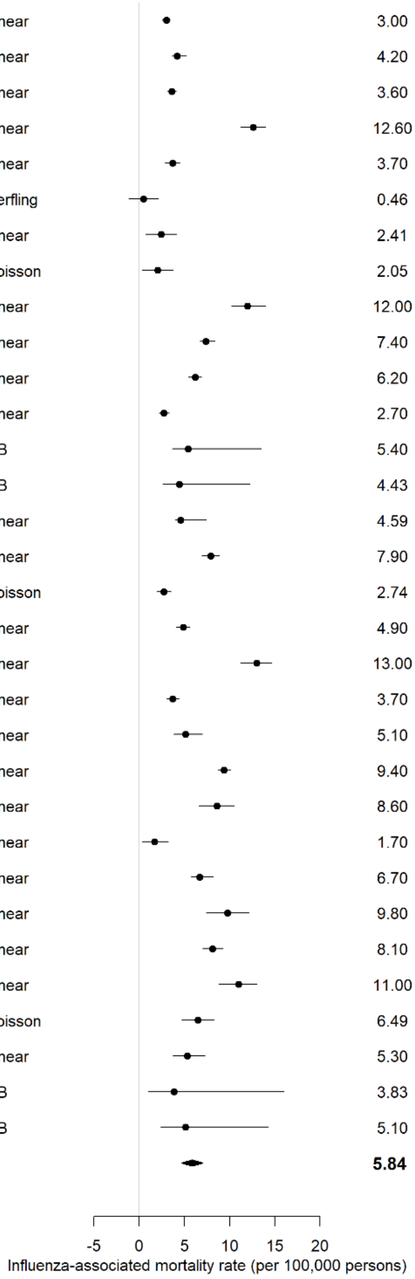

B. Age-standardized

Study	Region	Model	EMR	95% CI
Li, 2018	Heilongjiang	Linear	2.04	(1.02, 3.06)
Li, 2018	Liaoning	Linear	2.60	(1.34, 4.07)
Li, 2018	Jilin	Linear	2.72	(1.82, 3.64)
Li, 2018	Beijing	Linear	9.58	(6.95, 12.16)
Li, 2018	Tianjin	Linear	2.77	(1.20, 4.37)
Li, 2018	Gansu	Linear	8.97	(5.31, 12.47)
Li, 2018	Shandong	Linear	4.93	(3.50, 6.61)
Li, 2018	Shaanxi	Linear	4.56	(3.12, 6.11)
Li, 2018	Henan	Linear	1.97	(0.93, 3.02)
Yu, 2013	Northern rural sities	NB	6.50	(4.34, 16.19)
Yu, 2013	Northern urban sities	NB	4.03	(2.31, 11.33)
Li, 2018	Jiangsu	Linear	4.28	(2.20, 6.38)
Li, 2018	Anhui	Linear	3.12	(1.59, 4.58)
Li, 2018	Shanghai	Linear	11.02	(8.27, 13.75)
Li, 2018	Hubei	Linear	2.62	(1.36, 3.95)
Li, 2018	Sichuan	Linear	3.16	(0.17, 6.49)
Li, 2018	Zhejiang	Linear	6.20	(4.80, 7.60)
Li, 2018	Chongqing	Linear	5.02	(1.70, 8.37)
Li, 2018	Jiangxi	Linear	1.39	(-1.71, 4.58)
Li, 2018	Hunan	Linear	4.29	(1.87, 6.89)
Li, 2018	Guizhou	Linear	7.56	(3.16, 12.06)
Li, 2018	Fujian	Linear	6.17	(3.67, 8.72)
Li, 2018	Guangxi	Linear	8.34	(4.08, 12.51)
Guo, 2016	Zhuhai	Poisson	12.70	(10.51, 14.89)
Li, 2018	Guangdong	Linear	4.84	(1.54, 8.24)
Yu, 2013	Southern rural sities	NB	4.14	(1.03, 17.23)
Yu, 2013	Southern urban sities	NB	4.90	(2.30, 13.86)
**Overall (I^2=86.3%, p<0.001)**			**5.03**	**(3.91, 6.16)**

Note: Random-effects model

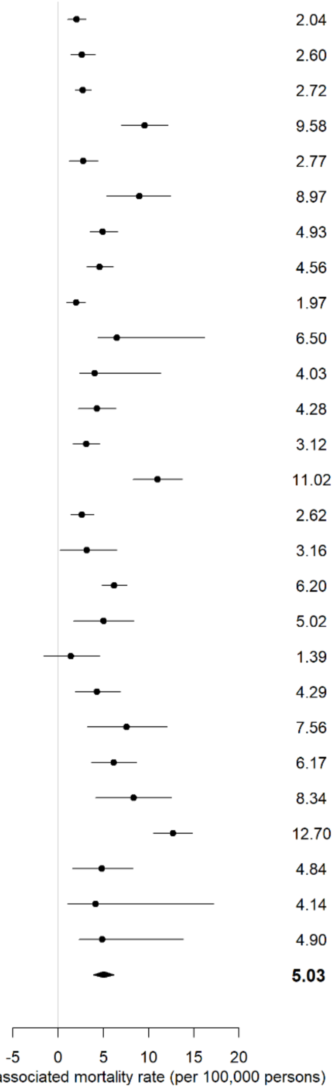

C. Age <65 years

Study	Region	Model	EMR	95% CI
Li, 2018	Heilongjiang	Linear	0.30	(-0.50, 1.10)
Li, 2018	Liaoning	Linear	0.60	(-0.50, 1.80)
Li, 2018	Jilin	Linear	1.10	(0.40, 1.80)
Li, 2018	Beijing	Linear	3.40	(1.30, 5.40)
Li, 2018	Tianjin	Linear	1.20	(0.00, 2.50)
Li, 2018	Gansu	Linear	1.70	(-1.10, 4.50)
Li, 2018	Shandong	Linear	1.40	(0.20, 2.70)
Li, 2018	Shaanxi	Linear	1.20	(0.10, 2.40)
Li, 2018	Henan	Linear	0.30	(-0.50, 1.10)
Yu, 2013	Northern rural sities	NB	1.00	(0.60, 2.73)
Yu, 2013	Northern urban sities	NB	0.50	(0.30, 2.28)
Li, 2018	Jiangsu	Linear	0.00	(-1.70, 1.70)
Li, 2018	Anhui	Linear	0.50	(-0.70, 1.70)
Li, 2018	Shanghai	Linear	8.40	(6.00, 10.80)
Li, 2018	Hubei	Linear	0.90	(-0.10, 2.00)
Li, 2018	Sichuan	Linear	0.80	(-1.80, 3.40)
Li, 2018	Zhejiang	Linear	1.20	(0.10, 2.30)
Li, 2018	Chongqing	Linear	1.20	(-1.70, 4.10)
Li, 2018	Jiangxi	Linear	0.30	(-2.00, 2.60)
Li, 2018	Hunan	Linear	0.60	(-1.40, 2.60)
Li, 2018	Guizhou	Linear	2.90	(-0.60, 6.40)
Li, 2018	Fujian	Linear	0.60	(-1.20, 2.50)
Li, 2018	Guangxi	Linear	2.90	(-0.30, 6.20)
Guo, 2016	Zhuhai	Poisson	1.55	(-0.34, 3.44)
Li, 2018	Guangdong	Linear	1.90	(-0.60, 4.30)
Yu, 2013	Southern rural sities	NB	0.40	(0.05, 2.23)
Yu, 2013	Southern urban sities	NB	0.63	(0.18, 2.78)
**Overall (I^2=53.6%, p=0.001)**			**1.23**	**(0.64, 1.82)**

Note: Random-effects model

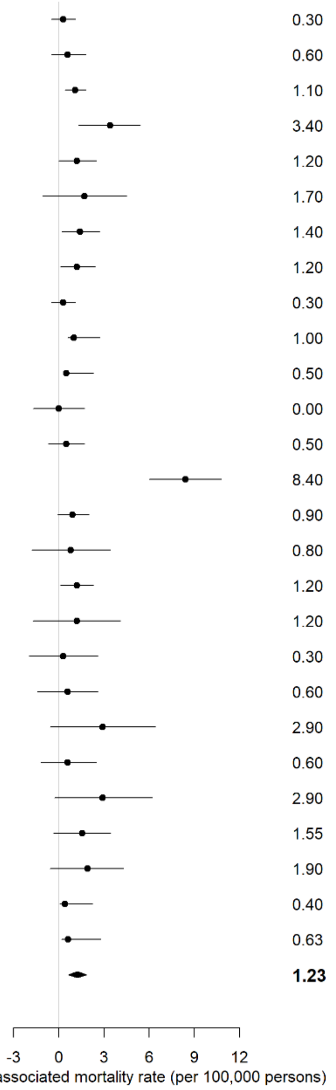

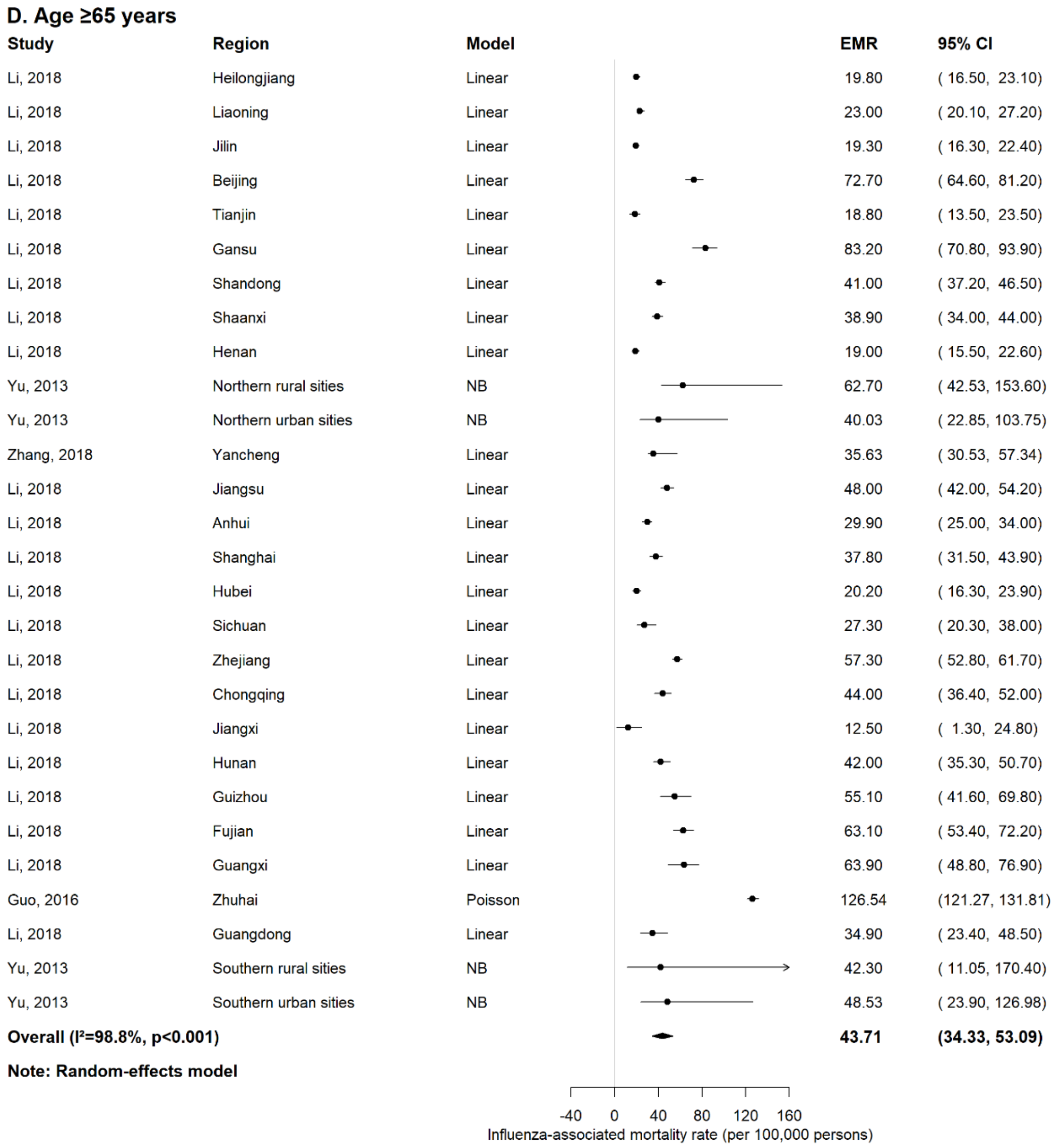

Figure S3. Age-specific influenza-associated respiratory mortality rates.

EMR, excess mortality rate; NB, negative binomial model.

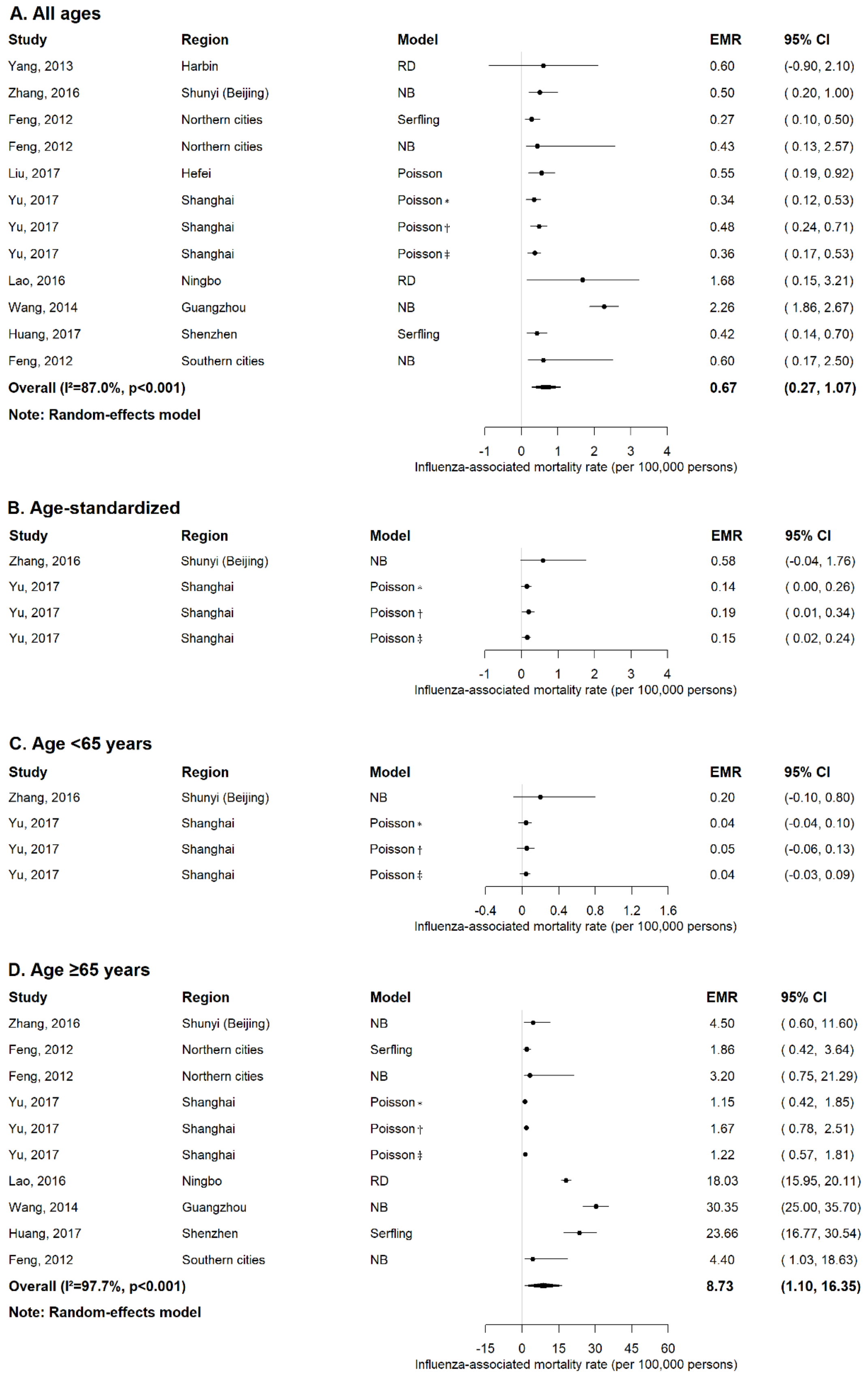
A. All ages
Study | Region | Model | EMR | 95% CI
Yang, 2013 | Harbin | RD | 0.60 | (-0.90, 2.10)
Zhang, 2016 | Shunyi (Beijing) | NB | 0.50 | (0.20, 1.00)
Feng, 2012 | Northern cities | Serfling | 0.27 | (0.10, 0.50)
Feng, 2012 | Northern cities | NB | 0.43 | (0.13, 2.57)
Liu, 2017 | Hefei | Poisson | 0.55 | (0.19, 0.92)
Yu, 2017 | Shanghai | Poisson * | 0.34 | (0.12, 0.53)
Yu, 2017 | Shanghai | Poisson † | 0.48 | (0.24, 0.71)
Yu, 2017 | Shanghai | Poisson ‡ | 0.36 | (0.17, 0.53)
Lao, 2016 | Ningbo | RD | 1.68 | (0.15, 3.21)
Wang, 2014 | Guangzhou | NB | 2.26 | (1.86, 2.67)
Huang, 2017 | Shenzhen | Serfling | 0.42 | (0.14, 0.70)
Feng, 2012 | Southern cities | NB | 0.60 | (0.17, 2.50)
Overall (I²=87.0%, p<0.001) | 0.67 | (0.27, 1.07)
Note: Random-effects model
-1 0 1 2 3 4
Influenza-associated mortality rate (per 100,000 persons)
B. Age-standardized
Study | Region | Model | EMR | 95% CI
Zhang, 2016 | Shunyi (Beijing) | NB | 0.58 | (-0.04, 1.76)
Yu, 2017 | Shanghai | Poisson * | 0.14 | (0.00, 0.26)
Yu, 2017 | Shanghai | Poisson † | 0.19 | (0.01, 0.34)
Yu, 2017 | Shanghai | Poisson ‡ | 0.15 | (0.02, 0.24)
-1 0 1 2 3 4
Influenza-associated mortality rate (per 100,000 persons)
C. Age <65 years
Study | Region | Model | EMR | 95% CI
Zhang, 2016 | Shunyi (Beijing) | NB | 0.20 | (-0.10, 0.80)
Yu, 2017 | Shanghai | Poisson * | 0.04 | (-0.04, 0.10)
Yu, 2017 | Shanghai | Poisson † | 0.05 | (-0.06, 0.13)
Yu, 2017 | Shanghai | Poisson ‡ | 0.04 | (-0.03, 0.09)
-0.4 0 0.4 0.8 1.2 1.6
Influenza-associated mortality rate (per 100,000 persons)
D. Age ≥65 years
Study | Region | Model | EMR | 95% CI
Zhang, 2016 | Shunyi (Beijing) | NB | 4.50 | (0.60, 11.60)
Feng, 2012 | Northern cities | Serfling | 1.86 | (0.42, 3.64)
Feng, 2012 | Northern cities | NB | 3.20 | (0.75, 21.29)
Yu, 2017 | Shanghai | Poisson * | 1.15 | (0.42, 1.85)
Yu, 2017 | Shanghai | Poisson † | 1.67 | (0.78, 2.51)
Yu, 2017 | Shanghai | Poisson ‡ | 1.22 | (0.57, 1.81)
Lao, 2016 | Ningbo | RD | 18.03 | (15.95, 20.11)
Wang, 2014 | Guangzhou | NB | 30.35 | (25.00, 35.70)
Huang, 2017 | Shenzhen | Serfling | 23.66 | (16.77, 30.54)
Feng, 2012 | Southern cities | NB | 4.40 | (1.03, 18.63)
Overall (I²=97.7%, p<0.001) | 8.73 | (1.10, 16.35)
Note: Random-effects model
-15 0 15 30 45 60
Influenza-associated mortality rate (per 100,000 persons)

Figure S4. Age-specific influenza-associated P&I mortality rates. P&I, pneumonia and influenza; EMR, excess mortality rate; RD, rate difference model; NB, negative binomial model. * Poisson regression model with LAB number for influenza activity proxy; † Poisson regression model with LAB% for influenza activity proxy; ‡ Poisson regression model with LAB%×ILI% for influenza activity proxy.

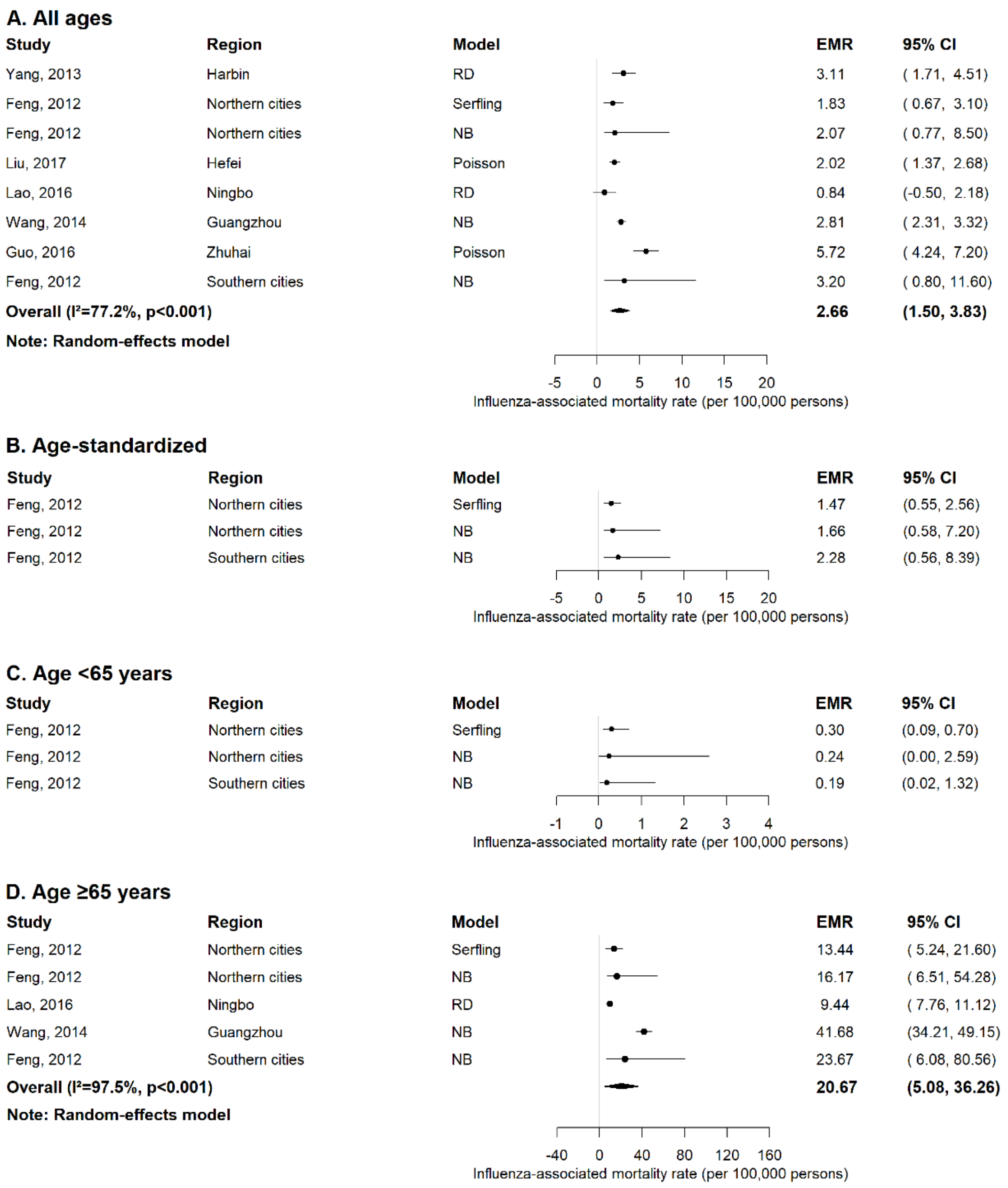

Figure S5. Age-specific influenza-associated COPD mortality rates. COPD, chronic obstructive pulmonary disease; EMR, excess mortality rate; RD, rate difference model; NB, negative binomial model.

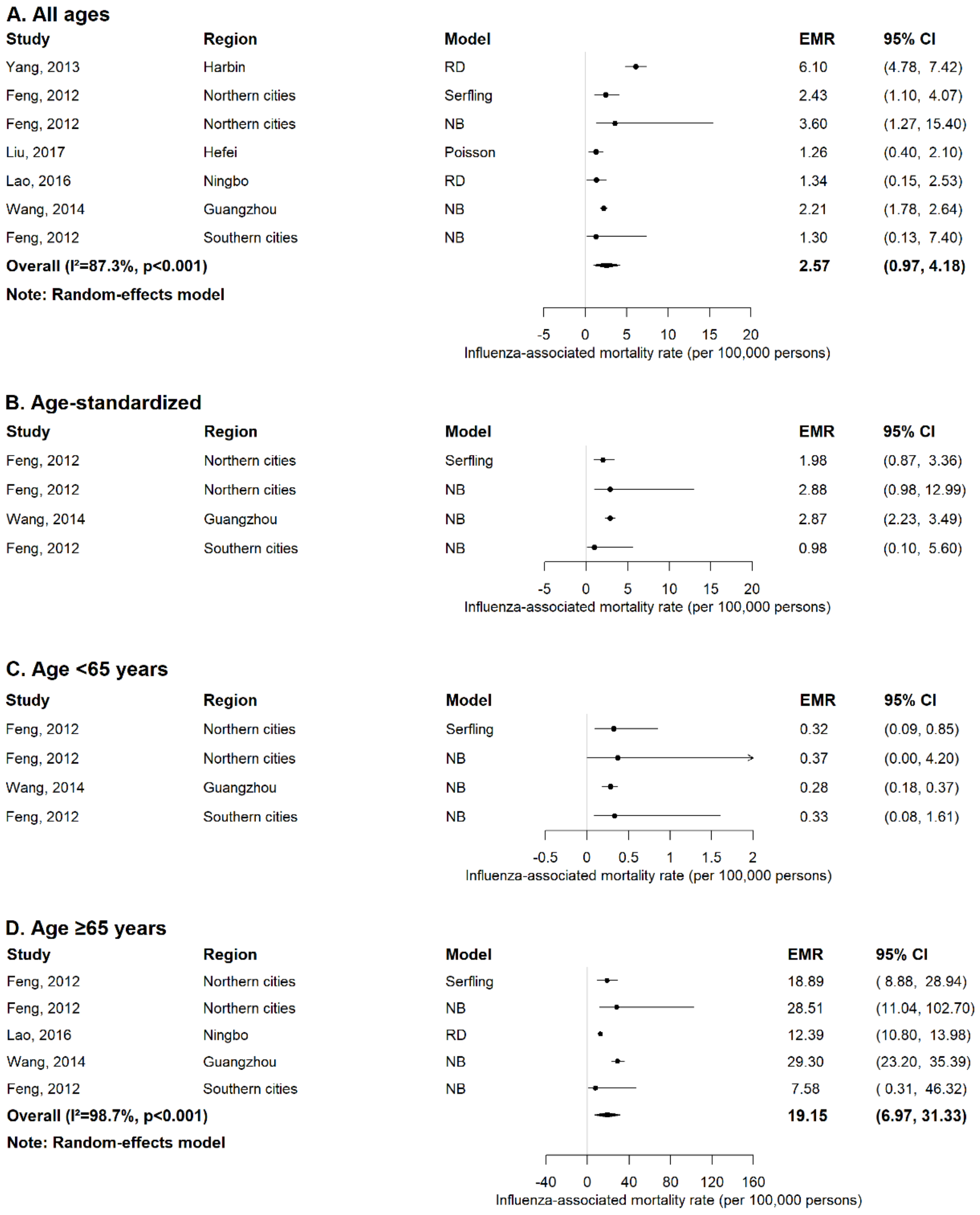

Figure S6. Age-specific influenza-associated IHD mortality rates. IHD, ischemic heart disease; EMR, excess mortality rate; RD, rate difference model; NB, negative binomial model.

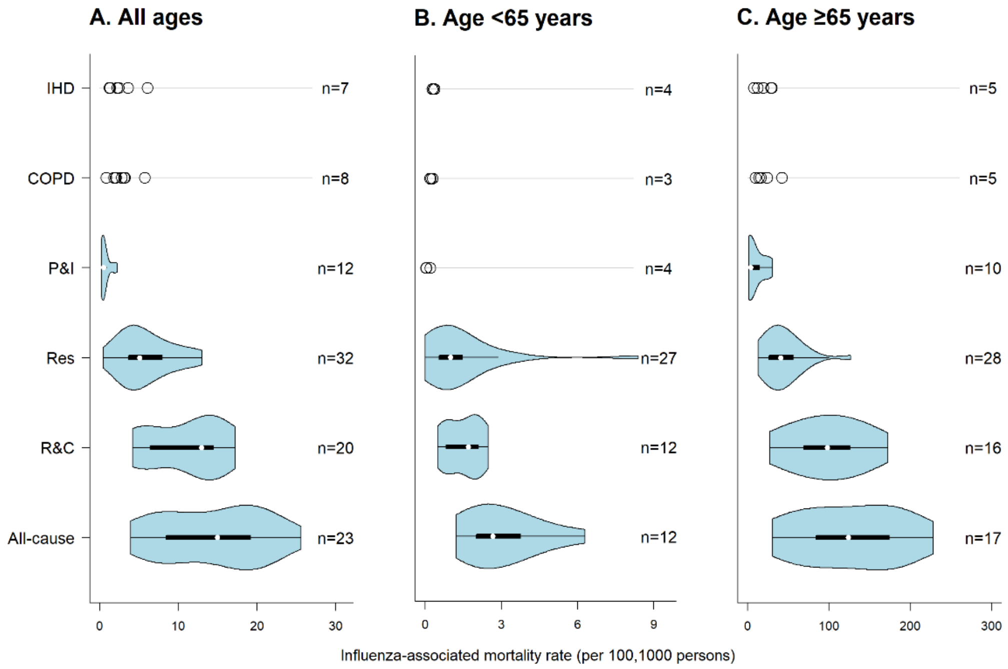

Figure S7. Estimates of annual influenza-associated mortality rates by age group and cause of death. IHD, ischemic heart disease; COPD, chronic obstructive pulmonary disease; P&I, pneumonia and influenza; Res, respiratory disease; R&C, respiratory and circulatory disease. If there were 10 or more studies reporting influenza-associated age- and cause-specific mortality rates, violin plots were plotted; otherwise only the annual average influenza-associated mortality rates from each included study were plotted.

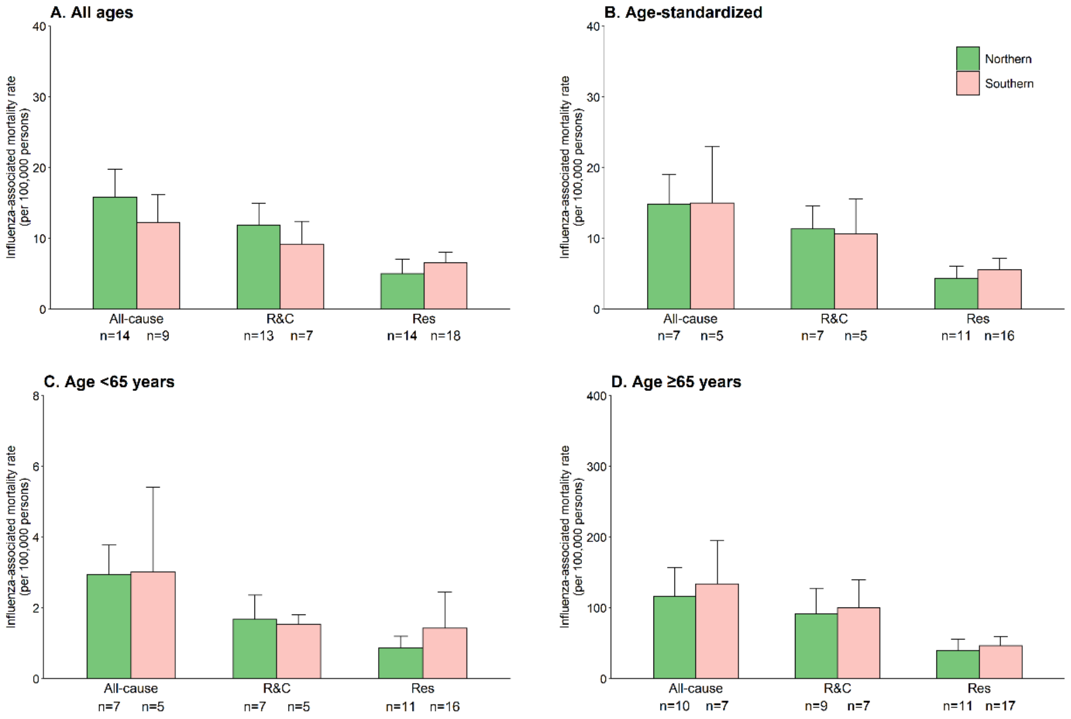

Figure S8. Pooled influenza-associated mortality rates by age group, cause of death, and geographic location. R&C, respiratory and circulatory disease; Res, respiratory disease.

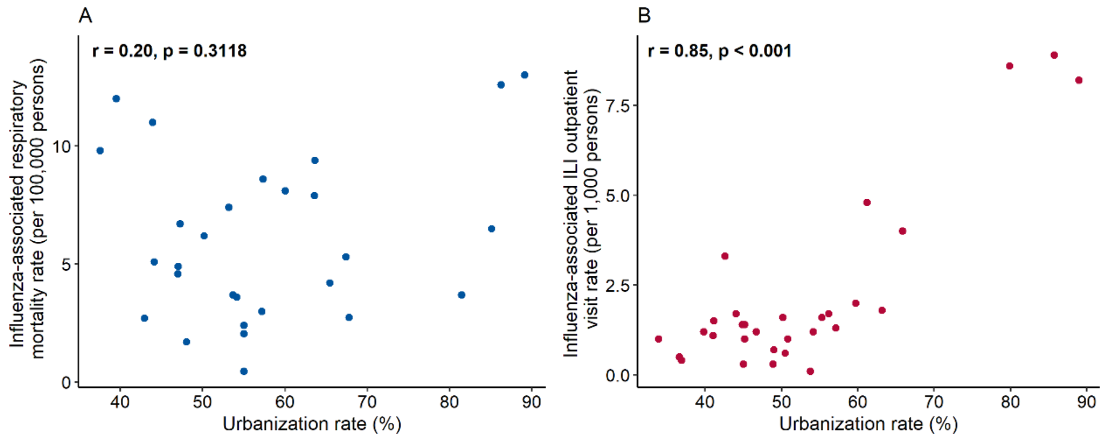

Figure S9. Correlations between urbanization rates and influenza associated respiratory mortality rates and ILI outpatient visit rates.

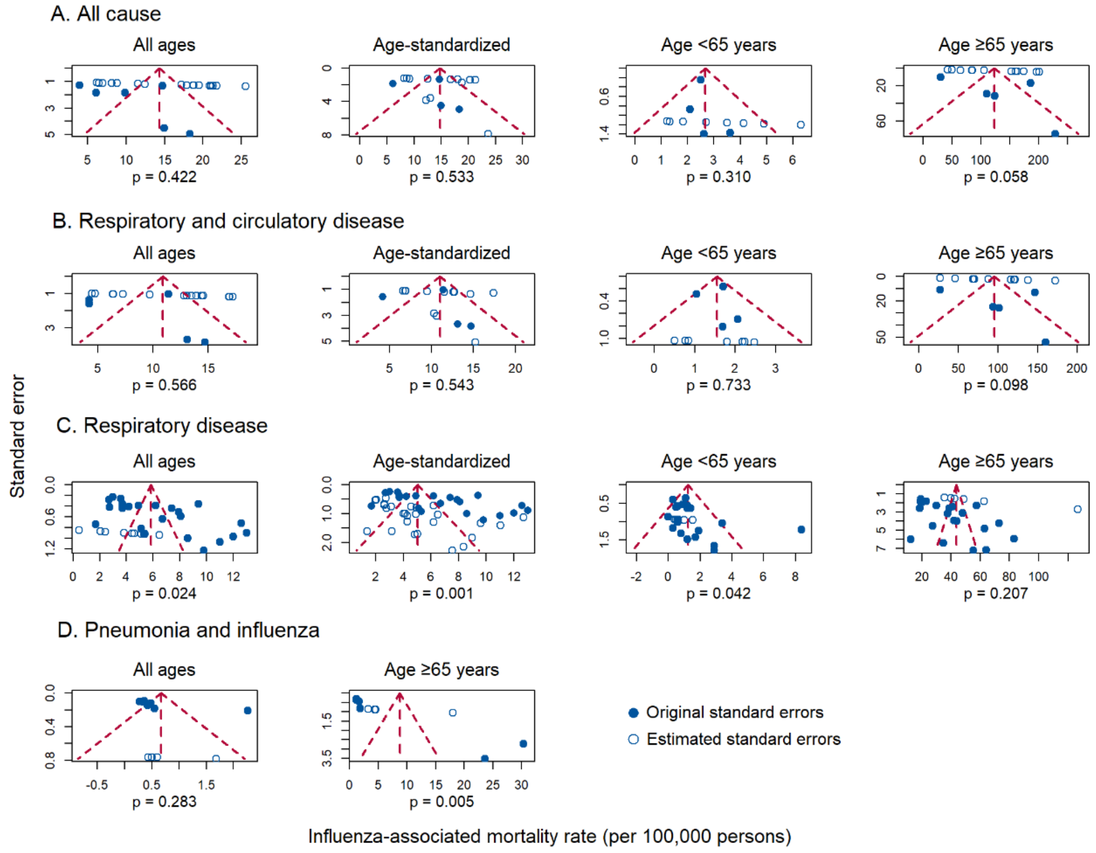

Figure S10. Funnel plot of influenza-associated mortality rates by cause of death and age group.

References

1 Chen, X. *et al.* Estimate of excess mortality attributed to influenza in Qingdao, 2001-2008. *Disease Surveillance* **25**, 289-293 (2010).

2 Yang, L. *et al.* Influenza associated mortality in the subtropics and tropics: results from three Asian cities. *Vaccine* **29**, 8909-8914, doi:10.1016/j.vaccine.2011.09.071 (2011).

3 Feng, L. *et al.* Influenza-associated mortality in temperate and subtropical Chinese cities, 2003–2008. *Bulletin of the World Health Organization* **90**, 279-288B, doi:10.2471/BLT.11.096958 (2012).

4 Yu, H. *et al.* Regional variation in mortality impact of the 2009 A(H1N1) influenza pandemic in China. *Influenza and other respiratory viruses* **7**, 1350-1360, doi:10.1111/irv.12121 (2013).

5 Yang, L. *et al.* Influenza-related excess mortality in Harbin, 2005-2009. *Chin J Public Health* **29**, 1031-1033 (2013).

6 Wang, H. *et al.* Influenza associated mortality in Southern China, 2010–2012. *Vaccine* **32**, 973-978, doi:https://doi.org/10.1016/j.vaccine.2013.12.013 (2014).

7 Li, S., Lin, H., Feng, L. & Yu, H. Estimates of influenza-associated excess mortality by two regression models in Dalian city during 1991-2008. *International Journal of Virology* **22**, 172-179, doi:10.3760/cma.j.issn.1673-4092.2015.03.008 (2015).

8 Guo, R.-n. *et al.* Impact of Influenza on Outpatient Visits, Hospitalizations, and Deaths by Using a Time Series Poisson Generalized Additive Model. *PLOS ONE* **11**, e0149468, doi:10.1371/journal.pone.0149468 (2016).

9 Zhang, W. *et al.* Influenza-associated excess mortality in Shunyi District, Beijing (2010-2015). *J of Pub Health and Pre Med* **27**, 26-30 (2016).

10 Lao, X., Jiao, S., Ji, W. & Yi, B. An analysis on the influenza-related excess mortality in Ningno City. *Pre Med* **28**, 1010-1013+1018 (2016).

11 Liu, X. X. *et al.* Excess mortality associated with influenza after the 2009 H1N1 pandemic in a subtropical city in China, 2010-2015. *International journal of infectious diseases : IJID : official publication of the International Society for Infectious Diseases* **57**, 54-60, doi:10.1016/j.ijid.2017.01.039 (2017).

12 Huang, Z. *et al.* Application of Serfling cyclical regression model in the estimation of influenza-associated excess mortality in Shenzhen. *Chin J Dis Control Prev* **21**, 1170-1174 (2017).

13 Yu, X. *et al.* Excess pneumonia and influenza mortality attributable to seasonal influenza in subtropical Shanghai, China. *BMC infectious diseases* **17**, 756-756, doi:10.1186/s12879-017-2863-1 (2017).

14 Zhang, H. *et al.* Influenza-associated mortality in Yancheng, China, 2011-15. **12**, 98-103, doi:doi:10.1111/irv.12487 (2018).

15 Wu, S. *et al.* Mortality burden from seasonal influenza and 2009 H1N1 pandemic influenza in Beijing, China, 2007-2013. *Influenza and other respiratory viruses* **12**, 88-97, doi:10.1111/irv.12515 (2018).

16 Li, L. *et al.* Influenza-associated excess respiratory mortality in China, 2010-15: a population-based study. *The Lancet Public Health* **4**, e473-e481, doi:http://dx.doi.org/10.1016/S2468-2667%2819%2930163-X (2019).

17 Zhao, M. *et al.* Estimates of influenza-associated excess mortality by three regression

models in Shanxi Province during 2013-2017. *Chin J Prev Med* **53**, 1012-1017, doi:10.3760/cma.j.issn.0253?9624.2019.10.011 (2019).
18 Feng, L. *et al.* Influenza-associated excess hospitalization in children, Wuxi city, Jiangsu province, 2005-2010. *Chin J Epidemiol*, 699-703, doi:10.3760/cma.j.issn.0254-6450.2014.06.018 (2014).
19 Zhang, X. *et al.* Pneumonia and influenza hospitalizations among children under 5 years of age in Suzhou, China, 2005-2011. *Influenza and other respiratory viruses* **11**, 15-22, doi:10.1111/irv.12405 (2017).
20 Wu, S. *et al.* Estimated incidence and number of outpatient visits for seasonal influenza in 2015-2016 in Beijing, China. *Epidemiol Infect* **145**, 3334-3344, doi:10.1017/s0950268817002369 (2017).
21 Zhang, X. *et al.* Estimated infection rates and incidence rates of seasonal influenza in Beijing during the 2017-2018 influenza season. *International Journal of Virology* **26**, 73-76, doi:10.3760/cma.j.issn.1673-4092.2019.02.001 (2019).
22 Feng, L. *et al.* Burden of influenza-associated outpatient influenza-like illness consultations in China, 2006-2015: A population-based study. *Influenza and other respiratory viruses*, doi:http://dx.doi.org/10.1111/irv.12711 (2019).
23 Ji, W. *et al.* The epidemiology of hospitalized influenza in children, a two year population-based study in the People's Republic of China. *BMC Health Services Research* **10**, 82, doi:10.1186/1472-6963-10-82 (2010).
24 Yu, H. *et al.* The substantial hospitalization burden of influenza in central China: surveillance for severe, acute respiratory infection, and influenza viruses, 2010-2012. *Influenza Other Respir Viruses* **8**, 53-65, doi:10.1111/irv.12205 (2014).
25 Zhao, X. *et al.* Study of characteristics and estimation of hospitalization rate for hospitalized cases of influenza in Huairou district. *International Journal of Virology* **25**, 281-285 (2018).
26 Zhang, Y. *et al.* Hospitalizations for Influenza-Associated Severe Acute Respiratory Infection, Beijing, China, 2014-2016. *Emerg Infect Dis* **24**, 2098-2102, doi:10.3201/eid2411.171410 (2018).
27 Yu, J. *et al.* Influenza-associated Hospitalization in Children Younger Than 5 Years of Age in Suzhou, China, 2011–2016. *The Pediatric Infectious Disease Journal* **38**, 445-452, doi:10.1097/inf.0000000000002178 (2019).
28 Zhang, W. *et al.* Hospitalization rates for influenza-associated severe acute respiratory illness in children younger than five years old in Suzhou of China, 2016-2018. *Chin J Prev Med* **53**, 1056-1059, doi:10.3760/cma.j.issn.0253?9624.2019.10.019 (2019).
29 Guo, R. N. *et al.* Epidemiologic and economic burden of influenza in the outpatient setting: a prospective study in a subtropical area of China. *PLoS One* **7**, e41403, doi:10.1371/journal.pone.0041403 (2012).
30 Gao, J. *et al.* The estimation of influenza-related outpatient rate in children under 5 years in Suzhou from 2011 to 2017. *Chin J Dis Control Prev* **23**, 34-38 (2019).
